# Supra-additive effect of chronic inflammation and atherogenic dyslipidemia on developing type 2 diabetes among young adults: a prospective cohort study

**DOI:** 10.1186/s12933-023-01878-5

**Published:** 2023-07-15

**Authors:** Yulong Lan, Dan Wu, Zhiwei Cai, Yuancheng Xu, Xiong Ding, Weiqiang Wu, Shaocong Lan, Lan Chen, Zheng Guo, Lois Balmer, Xingang Li, Manshu Song, Shouling Wu, Jingli Gao, Wei Wang, Youren Chen

**Affiliations:** 1 Centre for Precision Health, Edith Cowan University, Room 521, Building 21/270 Joondalup Drive, Perth, WA 6027 Australia; 2 Department of Cardiology, Second Affiliated Hospital of Shantou University Medical College, 69 Dongxia North Road, Shantou, 515041 China; 3 Department of Pediatrics, Second Affiliated Hospital of Shantou University Medical College, Shantou, 515041 China; 4 Department of Urology, The University of Hong Kong-Shenzhen Hospital, Shenzhen, 518172 China; 5 School of Public Health, Wuhan University, Wuhan, 430072 China; 6 Guangdong Medical University, Zhanjiang, 524023 China; 7 Department of Cardiology, The First Affiliated Hospital of Shantou University Medical College, Shantou, 515041 China; 8 Department of Cardiology, Kailuan General Hospital, Xinghua East Road, Tangshan, 063000 China; 9 Department of Intensive Care Unit, Kailuan General Hospital, Xinghua East Road, Tangshan, China; 10 School of Public Health, Shandong First Medical University & Shandong Academy of Medical Sciences, Tai’an, 271016 China; 11 Beijing Key Laboratory of Clinical Epidemiology, School of Public Health, Capital Medical University, Beijing, 100069 China

**Keywords:** Type 2 diabetes, Aging, Dyslipidemia, Inflammation, Young adults

## Abstract

**Background:**

Both elevated inflammation and atherogenic dyslipidemia are prominent in young-onset diabetes and are increasingly identified as biologically intertwined processes that contribute to diabetogenesis. We aimed to investigate the age-specific risks of type 2 diabetes (T2D) upon concomitant chronic inflammation and atherogenic dyslipidemia.

**Methods:**

Age-stratified Cox regression analysis of the risk of incident diabetes upon co-exposure to time-averaged cumulative high-sensitivity C-reactive protein (CumCRP) and atherogenic index of plasma (CumAIP) among 42,925 nondiabetic participants from a real-world, prospective cohort (Kailuan Study).

**Results:**

During a median 6.41 years of follow-up, 3987 T2D developed. Isolated CumAIP and CumCRP were significantly associated with incident T2D in the entire cohort and across all age subgroups. Both CumAIP and CumCRP were jointly associated with an increased risk of diabetes (*P*-interaction = 0.0126). Compared to CumAIP < -0.0699 and CumCRP < 1 mg/L, co-exposure to CumAIP ≥ − 0.0699 and CumCRP ≥ 3 mg/L had a significant hazard ratio (HR) [2.55 (2.23–2.92)] after adjusting for socio-demographic, life-style factors, family history of diabetes, blood pressure, renal function and medication use. The co-exposure-associated risks varied greatly by age distribution (*P*-interaction = 0.0193): < 40 years, 6.26 (3.47–11.28); 40–49 years, 2.26 (1.77–2.89); 50–59 years, 2.51 (2.00–3.16); 60–69 years, 2.48 (1.86–3.30); ≥ 70 years, 2.10 (1.29–3.40). In young adults (< 45 years), both exposures had a significant supra-additive effect on diabetogenesis (relative excess risk due to interaction: 0.80, 95% CI 0.10–1.50).

**Conclusions:**

These findings highlight the need for age-specific combined assessment and management of chronic inflammation and dyslipidemia in primary prevention against T2D, particularly for young adults. The clinical benefit derived from dual-target intervention against dyslipidemia and inflammation will exceed the sum of each part alone in young adults.

**Supplementary Information:**

The online version contains supplementary material available at 10.1186/s12933-023-01878-5.

## Background

The pandemic of type 2 diabetes (T2D) has become a serious public health threat [1–3]. A consistent downward trend in T2D onset age even worsens the situation [3, 4]. Mounting evidence has demonstrated that the younger the age at diabetes onset, the greater the risk of diabetes-related comorbidities, e.g., cardiovascular diseases (CVD) [5], diabetic kidney disease [6], dementia [7] and premature mortality [8]. The existence of “metabolic memory”, in which the influence of an early glycemic exposure environment is imprinted in target cells and organs and leads to prolonged impairments even after optimal glucose control [9], emphasizes the need for the clinical priority of early identification and intervention against risk factors for young-onset T2D.

Deeply involved lipid abnormalities and systemic inflammation, diabetogenesis is a chronic, multifactorial complex process. The atherogenic dyslipidemia complex, manifested as high triglyceride (TG) and low high-density lipoprotein cholesterol (HDL-C) levels, has been identified to predispose individuals to T2D onset [10, 11]. As such, the atherogenic index of plasma (AIP), measured as log-transformed (TG/HDL-C) [12], has been established to predict diabetes [13] and diabetic vascular complications [14–16]. Systemic inflammation, another hallmark of overt hyperglycemia, is also heavily engaged in diabetogenesis [17]. Indeed, both of them have been proposed to be closely entangled biological processes that can each amplify the other in an in vivo pathophysiologic setting, leading to impairment in insulin signaling [18, 19]. Notably, both abnormalities are probably linked to the aging process, mostly occurring after middle age [20–22], similar to the usual trend of T2D onset. Nevertheless, it is increasingly observed that young-onset diabetes has prominent dyslipidemia and systemic inflammation [4, 23, 24]. It is unclear whether these two disorders interact to affect young-onset diabetes. To date, limited epidemiological studies have examined the age-specific interactions between these two concomitant exposures and their joint effect on developing T2D among the general population.

In this context, studies examining the age-stratified risks of T2D with combined exposure to dyslipidemia and inflammation and their interactions throughout the adult lifespan are warranted. To fill in this knowledge gap, therefore, we conducted an analysis of datasets from a large, prospective cohort study (Kailuan study).

## Methods

### Study setting and study participants

The Kailuan Study (trial registration number: ChiCTR-TNC-11001489) is a large, ongoing, real-world, community-based cohort study in Tangshan, China. This study was initially carried out in 2006, with subsequent surveys issued every two years. Details of the study design and procedures have been specified elsewhere [25–28]. Written informed consent was provided before enrollment by each participant. This current subanalysis was approved by the Kailuan General Hospital Ethics Committee, China (2006-05) and the Human Research Ethics Committee of Edith Cowan University (2021-03159-BALMER).

For the present study, among 101510 participants who completed the first health survey, we excluded those who missed the following two examinations (n = 43,583); were diagnosed with diabetes (n = 8865); had incomplete information on fasting blood glucose (FBG), lipid profiles and high sensitivity C-reactive protein (hsCRP) (n = 2737) or abnormal values in these variables (n = 294); and those who missed all three follow-up visits through July 31, 2018 (n = 3106). Additional file 1: Fig. S1 displays the study strategy, and Additional file 1: Fig. S2 describes the flowchart of participants. The final analytic sample included 42,925 participants. The frequency and number of participants in follow-up are reported in Additional file 1: Table S1.

### Ascertainment of outcome

The primary study outcome was the incidence of T2D (ICD-10: E11), defined as either FBG level ≥ 7.0 mmol/L, self-reported history of a physician diagnosis, or self-reported medication use of oral anti-glycemic agents or insulin [29]. Death was ascertained from local government vital statistics offices [30]. The T2D onset date was defined as the first of the three follow-up surveys at which a participant met the diagnostic criteria. Follow-up ended at the date of T2D onset, death, or the last follow-up visit, whichever came first.

### Combined exposure to chronic inflammation and atherogenic dyslipidemia

As represented in Additional file 1: Fig. S1, the exposure period was from 2006/2007 to 2010/2011; the median exposure period was 3.92 [interquartile range (IQR): 3.87–4.32] years. Chronic inflammation was assessed by time-averaged cumulative hsCRP (CumCRP), calculated as [(hsCRP1 + hsCRP2)/2*(visit2 − visit1) + (hsCRP2 + hsCRP3)/2*(visit3 − visit2)]/(visit3-visit1) [31, 32]. Chronic atherogenic dyslipidemia, assessed by time-averaged cumulative AIP (CumAIP), was calculated using the abovementioned algorithm, with AIP = log10(TG/HDL-C) [12]. The study participants were stratified according to the median CumAIP level in this study and CumCRP cutoffs (1, 3 mg/L, the suggested clinical thresholds of hsCRP for indicating low, moderate metabolic inflammation [33]), thus creating six such subgroups: Group 1 (CumCRP < 1 mg/L and CumAIP < -0.0699), Group 2 (1 ≤ CumCRP < 3 mg/L and CumAIP < -0.0699), Group 3 (CumCRP ≥ 3 mg/L and CumAIP < -0.0699), Group 4 (CumCRP < 1 mg/L and CumAIP ≥ -0.0699), Group 5 (1 ≤ CumCRP < 3 mg/L and CumAIP ≥ -0.0699), and Group 6 (CumCRP ≥ 3 mg/L and CumAIP ≥ -0.0699).

### Covariates

Data on socio-demographics, lifestyle factors (drinking habit, smoking status, physical activities) and past medical and medication history (diabetes, CVD, hypertension, dyslipidemia, and current treatments including antihypertensives, antidiabetics and lipid-lowering agents) were documented with a standard questionnaire via face-to-face interviews. Biochemical measures of the lipid profiles, creatinine, FBG, and hsCRP were measured by an autoanalyzer (Hitachi 747; Hitachi, Tokyo, Japan). Anthropometrics, including blood pressure, height and weight, were collected. Body mass index (BMI) was calculated as weight (kilogram)/height^2^ (meter). Current smokers were defined as people who smoked at least one cigarette/day on average in the past year. Drinking status was defined according to average alcohol consumption in the past year. The estimated glomerular filtration rate (eGFR) was determined from creatinine following the Chronic Kidney Disease Epidemiology Collaboration formula [34]. Hypertension was stratified into four categories: normal blood pressure, grade I hypertension, grade II hypertension, and grade III hypertension [35].

### Statistical analyses

Baseline information was based on the commencement of follow-up. The included participants aged 22 to 98 years at baseline were categorized as follows: < 40 years, 40–49 years, 50–59 years, 60–69 years, and ≥ 70 years. We used multiple imputation by chained equation techniques to account for missing data (< 2% incomplete). Baseline characteristics were described as the mean with standard deviation (SD), median together with IQR, or numbers and percentages (%), when appropriate. Log-transformed values of CumCRP, hsCRP and TG were used when they were included as continuous variables in the statistical model. Baseline characteristics were compared by age group using one-way ANOVA or the Kruskal‒Wallis test for continuous variables and the chi-square test for categorical variables. The linear trend for individual factors across age groups was conducted by assigning the median value of each age group as a continuous variable in a separate model, utilizing a general linear model for continuous variables and a logistic model for categorical variables. *P* values for trends were calculated using the Wald test.

T2D incidence rates were calculated as per 1000 person-years. The Kaplan–Meier method with a log-rank test was performed to compare the overall survival based on follow-up intervals in each risk group. Stratified Cox proportional hazards regression models were used (except for single CumCRP exposure, where weighted Cox modeling was used because of the violation of the proportional hazards assumption) to calculate adjusted hazard ratios (aHRs) with 95% confidence intervals (CIs) of CumAIP and CumCRP, alone or as adjuncts, for incident T2D in the entire cohort and among age subgroups (stratified by < 40, 40 ~ 49, 50 ~ 59, 60 ~ 69, ≥ 70 years and stratified by < 45, 45 ~ 64, ≥ 65 years). The multivariable-adjusted models were as follows: Model 1, adjusted for age, sex, education, smoking and drinking status, physical activities, family history of diabetes, BMI, antihypertensives and lipid-lowering drugs; Model 2, further adjusted for eGFR, total cholesterol (TC), and blood pressure. Likelihood ratio tests evaluated the multiplicative interaction (INTm) between the combined exposures and age, between isolated CumAIP or CumCRP and age, and between CumAIP and CumCRP in the fully multivariable-adjusted Cox models. The relative excess risk due to interaction (RERI) was assessed as an index of additive interaction [36, 37] between elevated CumCRP (≥ 3 mg/L) and CumAIP (≥ median) in developing diabetes, with CumCRP < 3 mg/L and CumAIP < median as the baseline. Briefly, on the hazard ratio scale, we decomposed the joint excess relative risk for both exposures (HR11-1) into the excess relative risk for elevated CumAIP (HR01-1), elevated CumCRP (HR10-1), and relative excess risk due to interaction (RERI). Specifically, we have HR11 -1 = (HR01-1) + (HR10-1) + RERI [37].

To assess the robustness of the findings, sensitivity analyses were performed by additionally adjusting for fatty liver degree as fatty liver being a result of hepatic insulin resistance (IR), lipid and inflammation disorders [38], excluding T2D occurring within the first follow-up visit, excluding participants with known CVD, excluding those with impaired fasting glucose during the exposure period, and excluding participants with incomplete data.

All statistical analyses were performed with SAS software (version 9.4; SAS Institute, Cary, NC). A two-tailed *P* value < 0.05 was considered statistically significant, except for interaction testing, where a *P* value < 0.1 was considered significant. RERI and AP greater than zero were defined as a positive deviation and considered significant when the 95% CI did not contain zero.

## Results

Among the 42,925 individuals without preexisting diabetes (males [75.2%] and mean [SD] age, 52.1 [11.8] years), during a median 6.41 years (IQR: 4.25–6.87) of follow-up, 3,987 cases of T2D developed. Table 1 displays the characteristics of time-averaged cumulative and baseline information by age strata. For cumulative profiles, a persistently increasing tendency was observed in CumCRP with increasing age, whereas CumTG showed an inverse relationship. Cumulative HDL-C was slightly higher in middle age but declined in the older age group. In terms of baseline characteristics, systolic blood pressure, diastolic blood pressure, FBG, hsCRP, TC, LDL-C, HDL-C, creatine and BMI were positively associated with advancing age, whereas TG declined with advancing age. Additionally, elderly participants free of diabetes were less likely to be current smokers, current drinkers, or physically inactive and had a lower positive family history of diabetes and prevalence of dyslipidemia.

**Table 1:** Baseline characteristics of the study population

Characteristics	Total (n = 42,925)		< 40 years (n = 6364)		40–49 years (n = 11,521)		50–59 years (n = 13,923)		60–69 years (n = 7658)		≥ 70 years (n = 3459)		*P* for trend	
Cumulative characteristics														
CumCRP, mg/L	1.5 (0.8,3.0)		1.3 (0.7,2.4)		1.3 (0.7,2.5)		1.6 (0.9,3.1)		1.9 (1.0,3.7)		2.2 (1.1,4.3)		< 0.01	
CumAIP	− 0.06 ± 0.27		− 0.06 ± 0.27		− 0.05 ± 0.29		− 0.05 ± 0.26		− 0.07 ± 0.24		− 0.10 ± 0.22		< 0.01	
CumTG, mmol/L	1.3 (1.0,1.9)		1.4 (1.0,2.0)		1.4 (1.0,2.1)		1.4 (1.0,2.0)		1.2 (0.9,1.7)		1.1 (0.9,1.5)		< 0.01	
CumHDL, mmol/L	1.5 (1.3,1.8)		1.5 (1.4,1.7)		1.6 (1.4,1.8)		1.6 (1.3,1.8)		1.5 (1.3,1.7)		1.4 (1.2,1.7)		< 0.01	
Baseline characteristics														
Age, years	52.1 ± 11.8		33.4 ± 3.8		45.1 ± 2.7		54.5 ± 2.7		63.5 ± 2.9		74.6 ± 4.1		< 0.01	
AIP	− 0.06 ± 0.31		− 0.03 ± 0.32		− 0.05 ± 0.33		− 0.05 ± 0.31		− 0.09 ± 0.29		− 0.12 ± 0.27		< 0.01	
Male, no. (%)	32,290 (75.2)		4694 (73.8)		8340 (72.4)		10,452 (75.1)		5972 (78.0)		2832 (81.9)		< 0.01	
BMI, kg/m^2^	25.0 ± 3.3		24.7 ± 3.8		24.9 ± 3.2		25.0 ± 3.2		25.4 ± 3.3		24.7 ± 3.4		< 0.01	
SBP, mmHg	129.4 ± 18.6		119.2 ± 13.7		124.5 ± 15.9		130.8 ± 17.8		137.4 ± 19.6		141.4 ± 20.6		< 0.01	
DBP, mmHg	80.7 (79.3,90.0)		80.0 (71.5,85.0)		80.7 (78.7,90.0)		82.7 (80.0,90.0)		83.3 (80.0,90.7)		80.7 (80.0,90.0)		< 0.01	
FBG, mmol/L	5.2 ± 0.6		5.1 ± 0.6		5.2 ± 0.6		5.3 ± 0.6		5.3 ± 0.6		5.3 ± 0.6		< 0.01	
HDL-C, mmol/L	1.5 (1.3,1.8)		1.4 (1.2,1.8)		1.5 (1.2,1.8)		1.5 (1.2,1.8)		1.5 (1.3,1.9)		1.5 (1.3,1.9)		< 0.01	
LDL-C, mmol/L	2.6 ± 0.8		2.5 ± 0.7		2.6 ± 0.7		2.6 ± 0.8		2.6 ± 0.9		2.6 ± 0.9		< 0.01	
TC, mmol/L	5.0 ± 1.0		4.7 ± 0.9		4.9 ± 0.9		5.0 ± 1.0		5.1 ± 1.0		5.1 ± 1.0		< 0.01	
TG, mmol/L	1.3 (0.9,1.8)		1.3 (0.9,1.9)		1.3 (0.9,2.0)		1.3 (0.9,1.9)		1.2 (0.9,1.7)		1.1 (0.8,1.6)		< 0.01	
Creatine, umol/L	79.9 ± 21.1		78.4 ± 20.8		78.5 ± 20.3		79.5 ± 19.7		81.9 ± 19.3		84.9 ± 21.1		< 0.01	
HsCRP, mg/L	1.0 (0.5,2.4)		0.8 (0.3,1.9)		0.9 (0.4,2.2)		1.0 (0.5,2.3)		1.2 (0.7,2.7)		1.4 (0.8,3.2)		< 0.01	
Alcohol consumption, no. (%)														< 0.01
No		27,879 (64.9)		3700 (58.1)		6653 (57.7)		9082 (64.8)		5736 (74.9)		2762(79.8)		
Yes		15,046 (35.1)		2664 (41.9)		4868 (42.3)		4895 (35.2)		1922 (25.1)		697 (20.2)		
Smoking status, no. (%)														< 0.01
Never		26,418 (61.5)		3798 (59.7)		6522 (56.6)		8119 (58.3)		5329 (69.6)		2650 (76.6)		
Former		1943 (4.6)		233 (3.6)		466 (4.0)		650 (4.7)		409 (5.3)		185 (5.3)		
Current		14,564 (33.9)		2333 (36.7)		4533 (39.4)		5154 (37.0)		1920 (25.1)		624 (18.1)		
Education, no. (%)														< 0.01
Less than high school		32,554 (75.8)		2881 (45.3)		8682 (75.4)		11,843(85.1)		6464 (84.4)		2684 (77.6)		
High school and above		10,371 (24.2)		3483 (54.7)		2839 (24.6)		2080 (14.9)		1194 (15.6)		775 (22.4)		
Physical activity, no. (%)														< 0.01
Low		14,412 (33.5)		2370 (37.2)		4407 (38.3)		4774 (34.3)		1901 (24.8)		960 (27.8)		
Moderate		22,479 (52.4)		3508 (55.2)		6075 (52.7)		7204 (51.7)		4004 (52.3)		1688 (48.8)		
High		6034 (14.1)		486 (7.6)		1039 (9.0)		1945 (14.0)		1753 (22.9)		811 (23.4)		
Family history of diabetes		2290 (5.3)		561 (8.8)		837 (7.3)		703 (5.0)		162 (2.1)		27 (0.8)		< 0.01
Hypertension		20,630 (48.1)		1295 (20.3)		4559 (39.6)		7242 (52.0)		4985 (65.1)		2549 (73.7)		< 0.01
Dyslipidemia		11,691 (27.2)		1673 (26.3)		3196 (27.7)		3973 (28.5)		2021 (26.4)		828 (23.9)		< 0.01
Antihypertensives, no. (%)		2274 (5.3)		45 (0.7)		290 (2.5)		708 (5.1)		769 (10.0)		462 (13.4)		< 0.01
Statin, n (%)		251 (0.6)		16 (0.3)		47 (0.4)		81 (0.6)		70 (0.9)		37 (1.1)		< 0.01
Fibrate, n (%)		94 (0.2)		6 (0.1)		23 (0.2)		34 (0.2)		23 (0.3)		8 (0.2)		0.024

*CumAIP* cumulative atherogenic index for plasma, *CumCRP* cumulative high-sensitivity C-reactive protein, *CumHD*L cumulative high-density lipoprotein cholesterol, *CumTG* cumulative triglyceride, *DBP* diastolic blood pressure, *FBG* fasting blood glucose, *hsCRP* high-sensitivity C-reactive protein, *SBP* systolic blood pressure. Continuous variables with a normal distribution are presented as the mean with SD, while skewed data are presented as the median with IQR; categorical data are presented as numbers and percentages (%)

*P* for difference: all < 0.0001 except for Fibrate (*P* = 0.1128)

In the entire study population, isolated exposure to CumAIP (HR: 1.32, 95% CI 1.28–1.36 for per-SD increase in CumAIP) or CumCRP (average HR: 1.14, 95% CI 1.10–1.18 for per-SD increase in CumCRP) was significantly associated with incident diabetes after adjusting for age, sex, education level, smoking and drinking habits, physical activity, BMI, hypertension degree, renal function and medication use (Additional file 1: Tables S2–S3). The CumAIP-associated diabetic risks tended to be higher in those aged < 40 years (HR: 1.42, 95% CI: 1.27–1.57 per SD increase in CumAIP), albeit of a nonsignificant interaction. In contrast, a significant interaction was observed between isolated CumCRP and age groups [*P*-INTm: CumCRP cutoffs (1, 3 mg/L) × age groups: 0.0784; logCumCRP × age groups: 0.0027]. The average aHR (95% CI) per SD increase in logCumCRP was much higher in those aged < 40 years (HR: 1.32, 95% CI 1.18–1.48; *P*-trend: < 0.0001), gradually attenuating with each 10-year increase in follow-up age.

**Fig. 1:**
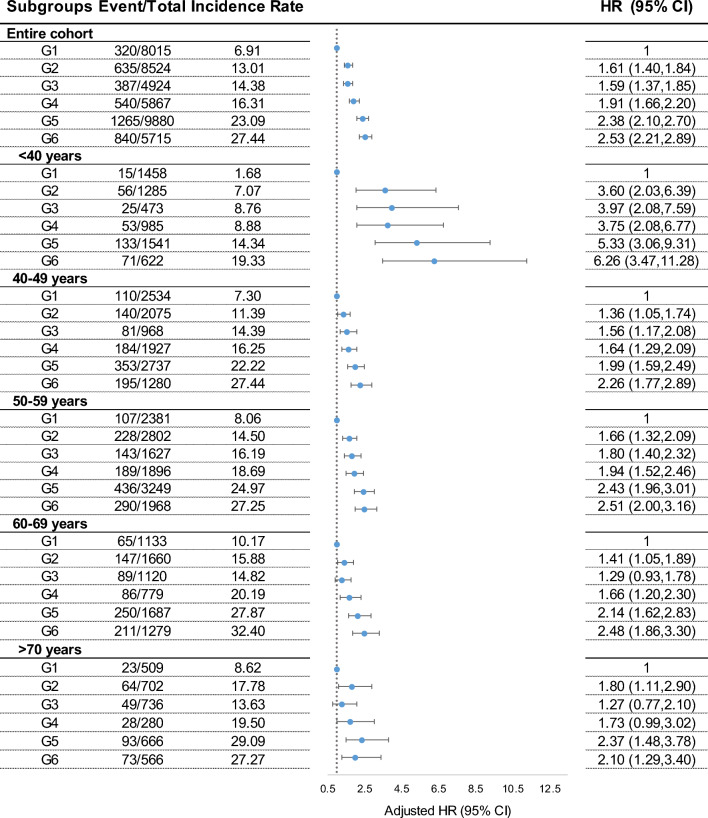
Age-associated risk of chronic inflammation and dyslipidemia for incident type 2 diabetes**.** *P*-INTm: Co-exposure subgroups and age-groups = 0.0193. G1: CumAIP < − 0.0699 and CumCRP < 1 mg/L; G2: CumAIP < -0.0699 and 1 ≤ CumCRP < 3 mg/L; G3: CumAIP < − 0.0699 and CumCRP ≥ 3 mg/L; G4: CumAIP ≥ − 0.0699 and CumCRP < 1 mg/L; G5: CumAIP ≥ − 0.0699 and 1 ≤ CumCRP < 3 mg/L; G6: CumAIP ≥ − 0.0699 and CumCRP ≥ 3 mg/L. All models were adjusted for age, sex, education, smoking status, drinking status, physical exercise, family history of diabetes, BMI, *TC* hypertension, *eGFR* antihypertensives (yes or no), and lipid-lowering agents (yes or no). Abbreviations as Table 1

The CumAIP-associated diabetes risks varied across different CumCRP strata (Additional file 1: Table S4). We then examined the association between co-exposure to CumAIP and CumCRP and incident type 2 diabetes. In the entire cohort, co-exposure was jointly associated with increased diabetic risks. A significant interaction between CumAIP and CumCRP was observed; CumAIP (median cut-point) * CumCRP thresholds (< 1, 1–3, ≥ 3 mg/L) = 0.0126). In those with CumAIP < -0.0699, individuals with an increasing CumCRP level had significantly higher aHRs (95% CIs) of 1.61 (1.41–1.85) and 1.59 (1.37–1.85) in the 1 ≤ CumCRP < 3 and CumCRP ≥ 3 mg/L strata, respectively, relative to CumCRP < 1 mg/L. In those with CumAIP ≥ -0.0699, elevation in CumCRP strata had markedly higher risks for incident diabetes (aHR [95% CI]: 1.91 [1.66–2.20], 2.38 [2.10–2.70] and 2.53 [2.21–2.89], respectively, in CumCRP < 1, 1 ≤ CumCRP < 3, CumCRP ≥ 3 mg/L). There was a significant risk difference in the co-exposure associated type 2 diabetes risks (*P-*INTm: Co- exposure subgroups* age groups (< 40, 40–49, 50–59, 60–69, ≥ 70 years) = 0.0193, Fig. 1). Additional file 1: Fig. S2 presents the Kaplan‒Meier curves of the cumulative incidence of T2D in the entire cohort and across the age subgroups. Compared to those with CumAIP < -0.0699 and CumCRP < 1 mg/L, the diabetic risks for CumAIP ≥ − 0.0699 and CumCRP ≥ 3 mg/L were markedly high in those aged < 40 years (HR: 6.26, 95% CI 3.47–11.28) and decreased dramatically with each 10 year increase in age (2.26 [95% CI 1.77–2.89] for 40–49 years, 2.51 [95% CI 2.00–3.16] for 50–59 years, 2.48 [95% CI 1.86–3.30] for 60–69 years, 2.10 [95% CI 1.29–3.40] for ≥ 70 years). The significant multiplicative interaction between CumCRP thresholds and CumAIP median persisted among those aged < 40 years (P-INTm: 0.0178; Additional file 1: Table S5). We then used different co-exposure categories as the reference group. As represented in Additional file 1: Tables S6–S8, for all age groups, participants in the same CumAIP stratum but with lower CumCRP levels had significantly decreased T2D risks; those with the same CumCRP level but with lower CumAIP levels also presented a significantly protective effect.

**Fig. 2:**
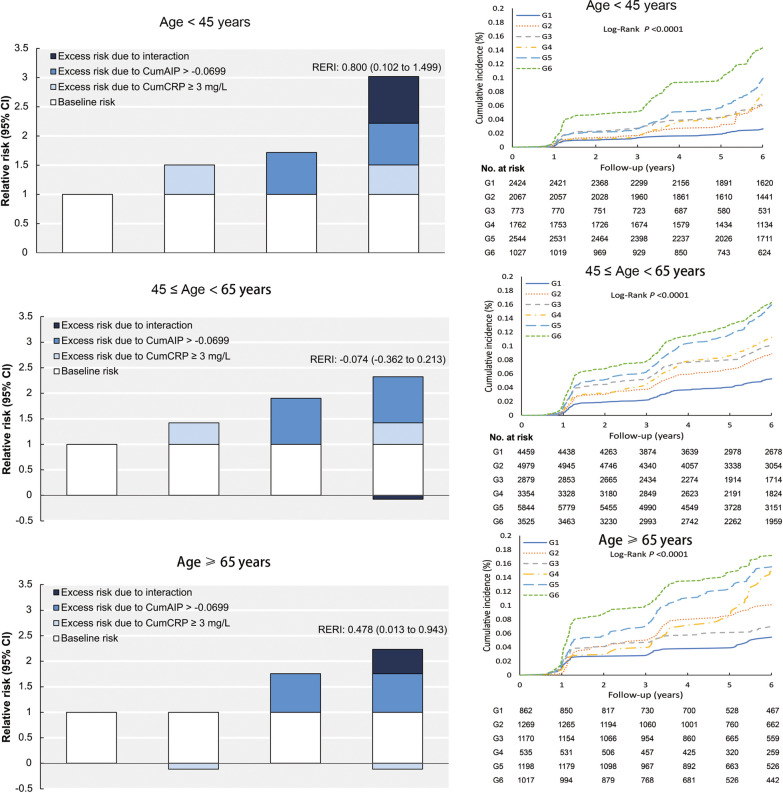
Kaplan‒Meier curves of the cumulative incidence of type 2 diabetes across age subgroups and age-specific relative risks of diabetes with separate contributions from chronic inflammation, atherogenic dyslipidemia, and their interaction**.** RERI: Relative excess risk due to interaction. For the analysis of RERI, the baseline category was low CumCRP (< 3 mg/L) with low CumAIP (< − 0.0699). Multi-variable Cox model was adjusted for sex, age, education, smoking status, drinking status, physical exercise, family history of diabetes, hypertension degrees, *TC* hypertension, *eGFR* antihypertensives, and lipid-lowering agents

We then investigated the co-exposure associated risk for incident diabetes with stratification of age < 45, 45–64, ≥ 65 years (Table 2). Likewise, there was a significant age heterogeneity (*P*-INTm = 0.0743). Co-exposure to CumAIP ≥ -0.0699 and CumCRP ≥ 3 mg/L was significantly associated with incident diabetes across age subgroups: 3.02 (95% CI 2.17–4.20) for the young (< 45 years), 2.39 (95% CI 2.03–2.81) for those aged 45 ~ 64 years, and 2.62 (95% CI 1.83–3.75) for the elderly (≥ 65 years). We specifically examined the supra-additive effect of elevated CumAIP and CumCRP across these age subgroups (Fig. 2**;** Additional file 1: Table S9). In the fully adjusted model, although a nonsignificant supra-addictive interaction was suggested among the entire cohort (RERI: 0.17, 95% CI − 0.06‒0.40) in the multivariable-adjusted model including socio-demographics, life-style factors, family history of diabetes, blood pressure, biological parameters and medication use, both elevations in CumCRP (≥ 3 mg/L) and CumAIP (≥ − 0.0699) levels reinforced a significant supra-addictive effect on diabetogenesis among the young (RERI: 0.80, 95% CI 0.10‒1.50; AP: 0.27, 95% CI 0.06‒0.47). Additionally, the supra-additive interaction between elevated CumAIP and CumCRP was borderline significant among the elderly (RERI: 0.48, 95% CI 0.01‒0.94; AP: 0.23, 95% CI 0.02‒0.43) but was nonsignificant among those aged 45 ~ 64 years (RERI: − 0.07, 95% CI − 0.36‒0.21; AP: − 0.03, 95% CI − 0.16‒0.10).

**Table 2:** Risk of incident type 2 diabetes with co-exposure to CumAIP and CumCRP across age subgroups (< 45, 45 – 65, ≥ 65 years)

Combination of CumCRP and CumAIP, HRs (95% CIs)						
	CumAIP < − 0.0699 and CumCRP < 1 mg/L	CumAIP < − 0.0699 and 1 ≤ CumCRP < 3 mg/L	CumAIP < − 0.0699 and CumCRP ≥ 3 mg/L	CumAIP ≥ − 0.0699 and CumCRP < 1 mg/L	CumAIP ≥ − 0.0699 and 1 ≤ CumCRP < 3 mg/L	CumAIP ≥ − 0.0699 and CumCRP ≥ 3 mg/L
< 45 years (702/10597)						
Incidence rate	3.91	8.68	9.62	11.18	15.16	23.31
Unadjusted model	Reference	2.22 (1.61,3.05)	2.47 (1.67,3.65)	2.81 (2.06,3.85)	3.82 (2.86,5.09)	5.98 (4.40,8.13)
Model 1	Reference	1.92 (1.39,2.64)	1.88 (1.27,2.78)	2.00 (1.45,2.76)	2.39 (2.77,3.23)	3.28 (2.36,4.55)
Model 2	Reference	1.90 (1.38,2.61)	1.83 (1.24,2.72)	1.85 (1.34,2.55)	2.21 (1.64,3.00)	3.02 (2.17,4.20)
*P*-INTm: CumAIP median × CumCRP cut-points (1, 3 mg/L) = 0.0439 (Model 2)						
45–65 years (2681/26277)						
Incidence rate	8.27	14.01	16.44	18.23	26.03	27.77
Unadjusted model	Reference	1.69 (1.43,1.99)	1.95 (1.64,2.33)	2.19 (1.85,2.58)	3.12 (2.69,3.62)	3.32 (2.84,3.87)
Model 1	Reference	1.53 (1.30,1.81)	1.69 (1.42,2.03)	1.90 (1.61,2.25)	2.46 (2.12,2.86)	2.47 (2.10,2.90)
Model 2	Reference	1.51 (1.29,1.78)	1.69 (1.42,2.03)	1.83 (1.55,2.17)	2.36 (2.03,2.75)	2.39 (2.03,2.81)
*P*-INTm: CumAIP median × CumCRP cut-points (1, 3 mg/L) = 0.0710 (Model 2)						
> 65 years (504/6051)						
Incidence rate	8.66	16.82	12.48	22.40	26.91	31.12
Unadjusted model	Reference	1.94 (1.35,2.78)	1.41 (0.96,2.08)	2.56 (1.72,3.80)	3.06 (2.17,4.33)	3.53 (2.49,5.00)
Model 1	Reference	1.80 (1.25,2.58)	1.28 (0.87,1.88)	2.20 (1.47,3.27)	2.45 (1.73,2.49)	2.71 (1.90,3.87)
Model 2	Reference	1.76 (1.22,2.53)	1.24 (0.84,1.83)	2.10 (1.41,3.13)	2.37 (1.66,3.37)	2.62 (1.83,3.75)
*P*-INTm: CumAIP median × CumCRP cut-points (1, 3 mg/L) = 0.0300 (Model 2)						

*P*-INTm: co-exposure subgroups*age subgroups (< 45, 45 ~ 65, ≥ 65 years) = 0.0743 (model 2)

Model 1: adjusted for age, sex, education, smoking status, drinking status, physical exercise, family history of diabetes, BMI, antihypertensives (yes or no), and lipid-lowering agents (yes or no);

Model 2: model 1 + TC, hypertension degree, and eGFR

*CumAIP* cumulative atherogenic index of plasma, *CumCRP* cumulative high-sensitivity C-reactive protein, *CI* confidence interval, *eGFR* estimated glomerular filtration rate, *HR* hazard ratio, *TC* total cholesterol, *INTm* multiplicative interaction

The results remained robust in the sensitivity analyses when additionally adjusting for the fatty liver degree **(**Additional file 1: Table S10), excluding study endpoints that occurred within the first follow-up visit **(**Additional file 1: Table S11), excluding participants with known CVD **(**Additional file 1: Table S12) or documented impaired fasting glucose in the exposure period **(**Additional file 1: Table S13), and the analysis was conducted on nonimputed data (Additional file 1: Tables S14–S15).

## Discussion

Our study observed a significant interaction between cumulative inflammation and atherogenic dyslipidemia associated with T2D onset among the general population. Moreover, this interaction was highly age dependent, particularly significant in young adults (age < 40 years). Young adults in this co-exposure had the highest adjusted HR (95% CI), attenuating greatly with increasing age. Notably, concomitant disorders in chronic inflammation and dyslipidemia had a synergistically additive effect on developing young-onset diabetes.

Our results provide support for the established biological link between atherogenic dyslipidemia and systemic inflammation in diabetogenesis [18] from an epidemiological landscape. It is essential to know that these risk factors do not act in isolation. Prior studies have demonstrated that these two systems are intertwined biological processes underlying the foundation of IR and insulin deficiency [10, 11, 39]. Prominent atherogenic dyslipidemia, probably as a result of obesity, enhances chronic tissue inflammation by elevating lipolysis and leakage of cytokines, thereby inducing IR [18, 40]. In turn, elevated inflammation and IR drive the remodeling of lipid fractions and compositions [41, 42], to an extent, worsening insulin signaling [10, 43], e.g., in obesity settings [40, 41].

Notably, in our study, the interaction between atherogenic dyslipidemia and inflammation and the T2D risk with their co-exposure were highly age specific. In co-exposure to chronic inflammation and atherogenic dyslipidemia, the T2D risks were markedly higher in young individuals (HR: 6.26, 95% CI [3.47–11.28]) than in other age groups. Moreover, the significant additive interaction between chronic inflammation and dyslipidemia in developing young-onset diabetes warrants attention, as the additive effect appeared to suggest a biological interplay, thus meriting further trials to unravel the underlying mechanism.

We interpreted the finding as the involvement of multifactorial influences on both lipid and inflammation abnormalities among young adults [3, 44]. As T2D is a typically age-related disease, its etiology is highly involved in “inflamm-aging” [45], where chronic, low-grade, and sterile inflammation accumulates with advancing age [20, 21]. Our findings that both cumulative and baseline hsCRP increased with every ten-year increase further corroborated this phenomenon. However, for reasons not completely understood, abnormal inflammation occurs early in young people and contributes to diabetogenesis. As such, young individuals who naturally have a low inflammation level but suffer from aberrant inflammation have a significantly higher diabetic risk, especially in those with basal metabolic disorders.

As described above [18, 40], the pandemic of overweight and obesity potentiated an important source of aberrant inflammation and atherogenic dyslipidemia, thus contributing to the magnitude of prevalence of young-onset diabetes [44, 46]. Aside from obesity-derived metabolic inflammation, pro-inflammatory diet preferences [47, 48], increased stress and depressive symptoms [49, 50] are also capable of enhancing the inflammatory burden in developing younger-onset diabetes. Indeed, impaired glucose control in younger adults was identified to be associated with biological and phycological factors, e.g., less healthy diet choices and habitual physical activities, negative life events, greater chronic stress, and higher depressed affect [51].

The significant age difference in the risk of developing diabetes conferred by the most common risk factors observed here partly explains the increasing prevalence of early-onset diabetes in current dyslipidemia- and inflammation-saturated environments. Young-onset diabetes is a unique patient subgroup due to its great etiological engagement in physical, psychological disorders, or both and needs special attention [51]. Additionally, younger-onset T2D is more detrimental to overall health [5–8], highlighting the significance of early precise intervention strategies against its onset. Convincing results from famous studies brought inspiring perspectives that early intensive interventions could reverse the risk of T2D [52, 53]. Our study supports intensive lifestyle management against atherogenic dyslipidemia for T2D prevention in all age groups. Furthermore, there is an urgent need for measures to identify and manage risk factors for excessively high inflammation in young adults. Notably, most chronic inflammation-promoting factors are probably modifiable, e.g., poor diet, sleep deficiency, physical inactivity, tobacco smoking, environmental toxicant exposure and psychological stress [54]. More social and family attention to both physical and psychological health in the young population is necessary [51].

This study has several strengths. The prolonged exposure burden is what truly matters in diabetogenesis [52]. With the facilitation of a longitudinal cohort, our study characterized the age-specific cumulative profiles in atherogenic lipid complexes and inflammation at approximately four years predating follow-up, which would be more stable and reliable than risk prediction tools based on cross-sectional data. Additionally, this cohort provided an opportunity to investigate the age-related risk factors for T2D in adulthood owing to an extensive time span of follow-up age. Furthermore, the high-quality data from this well-designed, prospective cohort study strengthened the weight of the results.

This study also has limitations. First, this investigation was composed primarily of Han Chinese individuals in North China, potentially limiting the generalizability of the results to individuals in the rest of the country or other races/ethnicities. Second, the sex skew of this cohort may lead to bias in the results, although it would be mild in view of the large study population. Third, there may have been biased estimations of the incidence of T2D, being overestimated as the diagnostic criteria were based on a single measurement of FBG instead of two test results or being underestimated as the diagnostic criteria were based on FBG level and/or antidiabetic medication, without the measurement of hemoglobin A1c and/or oral glucose tolerance testing [29]. Fourth, there may have been recall bias on covariates, including life-style factors (including drinking habits, smoking status and physical activities) and medication history, as they were self-reported from the participants [55]. This may partly explain the low prevalence of medication use of the study participants, as answering these questions naturally tends to be underreported, although the low prevalence of existing cardiometabolic diseases among the study participants and the region- and nation-specific differences in drug resources and policies as well as patient compliance in that era may also contribute to the low prevalence of medication use of anti-hypertensive and lipid-lowering drugs. Fifth, we had no available data regarding anti-inflammatory drugs among the study population, which may have potentially confounded the results. Additionally, we failed to distinguish between T2D and type 1 diabetes in this study cohort. Nevertheless, the misclassification would be minimal, given that the average age of the study population was much higher (52.1 years) than the usual type 1 diabetes onset age, and T2D accounts for 95% of all diabetes in the Chinese population. Further studies are warranted to replicate our findings in other populations.

## Conclusions

Our study, for the first time, demonstrated a significant age-dependent association of co-exposure to cumulative inflammation and atherogenic dyslipidemia with T2D onset among the Han Chinese population. In light of the significant interactions, it is promising to further leverage the associations to identify persons at risk of T2D and formulate age-specific, early, and active preventive strategies. Furthermore, it is reasonable to hypothesize that the clinical benefit derived from dual-target intervention against dyslipidemia and inflammation will exceed the sum of each part alone, especially in young adults.

## Supplementary Information

**Additional file 1: Table S1.** Numbers of participations in the follow-up period. Abbreviation: *No*. number. **Table S2.** Age-associated risk of CumAIP (quartiles) for type 2 diabetes. **Table S3.** Age-associated risk of CumCRP thresholds (1,3 mg/L) for type 2 diabetes. **Table S4.** CumAIP-associated type 2 diabetes risks across different CumCRP strata (<1,1–3,≥3 mg/L). **Table S5.** Age-associated risk of co-exposure to CumAIP and CumCRP for incident type 2 diabetes. **Table S6.** Age-associated risk of co-exposure to CumCRP and CumAIP for type 2 diabetes (reference group: CumAIP<− 0.0699 and 1≤ CumCRP <3 mg/L). **Table S7.** Age-associated risk of co-exposure to CumCRP and CumAIP for type 2 diabetes (reference group: CumAIP≥− 0.0699 and CumCRP≥3 mg/L). **Table S8.** Age-associated risk of co-exposure to CumCRP and CumAIP for type 2 diabetes (reference group: CumAIP≥− 0.0699 and 1≤CumCRP<3 mg/L). Table S9. Additive effect of elevated chronic inflammation and dyslypidemia on developing type 2 diabetes. **Table S10.** Sensitivity analysis of age-associated risks of co-exposure of CumCRP and CumAIP for type 2 diabetes by additional adjustment for baseline fatty liver degree (3987/42925). **Table S11.** Reverse analysis of age-associated risk of co-exposure to CumCRP and CumAIP for type 2 diabetes by excluding diabetes onset within the first follow-up visit (2492/41430). **Table S12.** Sensitivity analysis of age-associated risks of co-exposure of CumCRP and CumAIP for type 2 diabetes by excluding baseline CVD (3719/40713). **Table S13.** Sensitivity analysis of age-associated risks of co-exposure of CumCRP and CumAIP for type 2 diabetes by excluding impaired fasting glucose in exposure period (2061/35287). **Table S14.** Sensitivity analysis of age (<40,40–49,50–59,60–69,≥70)-associated risks of co-exposure of CumCRP and CumAIP for type 2 diabetes on raw data (3977/42807). **Table S15.** Sensitivity analysis of age(<45, 45–64,≥65)-associated risks of co-exposure of CumCRP and CumAIP for type 2 diabetes on raw data (3977/42807). **Fig S1.** Strategy of the study design. **Fig S2.** Flowchart of the study participants. **Fig S3.** Kaplan‒Meier curves of cumulative incidence of type 2 diabetes with the co-exposure subgroups in the entire cohort and across age subgroups

## Data Availability

The datasets used and/or analyzed during the current study are available from the corresponding author on reasonable request.

## Abbreviations

aHR: Adjusted hazard ratio
AIP: Atherogenic index of plasma
BMI: Body mass index
CIs: Confidence intervals
CumAIP: Cumulative atherogenic index of plasma
CumCRP: Cumulative high-sensitivity C-reactive protein
eGFR: Estimated glomerular filtration rate, FBG: fasting blood glucose
HDL-C: High-density lipoprotein cholesterol
HR: Hazard ratio
hsCRP: High sensitivity C-reactive protein
INTm: Multiplicative interaction
IQR: Interquartile range
IR: Insulin resistance
RERI: Relative excess risk due to interaction
SD: Standard deviation
TC: Total cholesterol
TG: Triglyceride
