# Supplementary material for: Supra-additive effect of chronic inflammation and atherogenic dyslipidemia on developing type 2 diabetes among young adults: a prospective cohort study
Source: Cardiovasc Diabetol. 2023 Jul 15;22:181. doi: 10.1186/s12933-023-01878-5 (PMC10350274; doi:10.1186/s12933-023-01878-5)
Supplement: Supplementary file 1 — Additional file 1: Table S1. Numbers of participations in the follow-up period. Abbreviation: No. number. Table S2. Age-associated risk of CumAIP (quartiles) for type 2 diabetes. Table S3. Age-associated risk of CumCRP thresholds (1,3 mg/L) for type 2 diabetes. Table S4. CumAIP-associated type 2 diabetes risks across different CumCRP strata (<1,1–3,≥3 mg/L). Table S5. Age-associated risk of co-exposure to CumAIP and CumCRP for incident type 2 diabetes. Table S6. Age-associated risk of co-exposure to CumCRP and CumAIP for type 2 diabetes (reference group: CumAIP<− 0.0699 and 1≤ CumCRP <3 mg/L). Table S7. Age-associated risk of co-exposure to CumCRP and CumAIP for type 2 diabetes (reference group: CumAIP≥− 0.0699 and CumCRP≥3 mg/L). Table S8. Age-associated risk of co-exposure to CumCRP and CumAIP for type 2 diabetes (reference group: CumAIP≥− 0.0699 and 1≤CumCRP<3 mg/L). Table S9. Additive effect of elevated chronic inflammation and dyslypidemia on developing type 2 diabetes. Table S10. Sensitivity analysis of age-associated risks of co-exposure of CumCRP and CumAIP for type 2 diabetes by additional adjustment for baseline fatty liver degree (3987/42925). Table S11. Reverse analysis of age-associated risk of co-exposure to CumCRP and CumAIP for type 2 diabetes by excluding diabetes onset within the first follow-up visit (2492/41430). Table S12. Sensitivity analysis of age-associated risks of co-exposure of CumCRP and CumAIP for type 2 diabetes by excluding baseline CVD (3719/40713). Table S13. Sensitivity analysis of age-associated risks of co-exposure of CumCRP and CumAIP for type 2 diabetes by excluding impaired fasting glucose in exposure period (2061/35287). Table S14. Sensitivity analysis of age (<40,40–49,50–59,60–69,≥70)-associated risks of co-exposure of CumCRP and CumAIP for type 2 diabetes on raw data (3977/42807). Table S15. Sensitivity analysis of age(<45, 45–64,≥65)-associated risks of co-exposure of CumCRP and CumAIP for type 2 diabetes on raw data (397 [file 12933_2023_1878_MOESM1_ESM.docx]

**Additional file Tables and Figures**

[Table S1. Numbers of participations in the follow-up period 2](#_Toc134278869)

[Table S2. Age-associated risk of CumAIP (quartiles) for type 2 diabetes 3](#_Toc134278870)

[Table S3. Age-associated risk of CumCRP thresholds (1,3 mg/L) for type 2 diabetes 5](#_Toc134278871)

[Table S4. CumAIP-associated type 2 diabetes risks across different CumCRP strata (<1,1-3,≥3 mg/L) 7](#_Toc134278872)

[Table S5. Age-associated risk of co-exposure to CumAIP and CumCRP for incident type 2 diabetes 8](#_Toc134278873)

[Table S6. Age-associated risk of co-exposure to CumCRP and CumAIP for type 2 diabetes (reference group: CumAIP<-0.0699 & 1≤ CumCRP <3mg/L) 10](#_Toc134278874)

[Table S7. Age-associated risk of co-exposure to CumCRP and CumAIP for type 2 diabetes (reference group: CumAIP≥-0.0699 & CumCRP≥3 mg/L) 11](#_Toc134278875)

[Table S8. Age-associated risk of co-exposure to CumCRP and CumAIP for type 2 diabetes (reference group: CumAIP≥-0.0699 & 1≤CumCRP<3 mg/L) 12](#_Toc134278876)

[Table S9. Additive effect of elevated chronic inflammation and dyslypidemia on developing type 2 diabetes 13](#_Toc134278877)

[Table S10. Sensitivity analysis of age-associated risks of co-exposure of CumCRP and CumAIP for type 2 diabetes by additional adjustment for baseline fatty liver degree (3987/42925) 15](#_Toc134278878)

[Table S11. Reverse analysis of age-associated risk of co-exposure to CumCRP and CumAIP for type 2 diabetes by excluding diabetes onset within the first follow-up visit (2492/41430) 17](#_Toc134278879)

[Table S12. Sensitivity analysis of age-associated risks of co-exposure of CumCRP and CumAIP for type 2 diabetes by excluding baseline CVD (3719/40713) 18](#_Toc134278880)

[Table S13. Sensitivity analysis of age-associated risks of co-exposure of CumCRP and CumAIP for type 2 diabetes by excluding impaired fasting glucose in exposure period (2061/35287) 19](#_Toc134278881)

[Table S14. Sensitivity analysis of age (<40,40-49,50-59,60-69,≥70)-associated risks of co-exposure of CumCRP and CumAIP for type 2 diabetes on raw data (3977/42807) 20](#_Toc134278882)

[Table S15. Sensitivity analysis of age(<45, 45-64,≥65)-associated risks of co-exposure of CumCRP and CumAIP for type 2 diabetes on raw data (3977/42807) 21](#_Toc134278883)

[Fig. S1 Strategy of the study design 22](#_Toc134278884)

[Fig. S2 Flowchart of the study participants 22](#_Toc134278885)

# Table S1. Numbers of participations in the follow-up period

| **No. of participation** | **No. of participants (42,925)** |
| --- | --- |
| 1 | 6,799 |
| 2 | 14,062 |
| 3 | 22,064 |

# Table S2. Age-associated risk of CumAIP (quartiles) for type 2 diabetes

|  | **CumAIP, HRs (95% CIs)** | | | | **Per SD^*^** | ***P* for trend** |  |
| --- | --- | --- | --- | --- | --- | --- | --- |
|  | **Quartile1** | **Quartile2** | **Quartile3** | **Quartile4** |  |  |  |
| Entire cohort | | | | | | |  |
| Event/Total | 538/10732 | 804/10731 | 1093/10730 | 1552/10732 |  |  |  |
| Incidence rate | 7.70 | 13.37 | 18.31 | 26.39 |  |  |  |
| Model 1 | Reference | 1.34 (1.20,1.50) | 1.70 (1.54,1.89) | 2.30 (2.08, 2.55) | 1.34 (1.30,1.38) | <0.0001 |  |
| Model 2 | Reference | 1.33 (1.19,1.48) | 1.67 (1.51,1.86) | 2.22 (2.00,2.46) | 1.32 (1.28,1.36) | <0.0001 |  |
| <40 years | | | | | | |  |
| Event/Total | 37/1621 | 59/1595 | 90/1548 | 1671600 |  |  |  |
| Incidence rate | 3.71 | 6.08 | 9.61 | 17.48 |  |  |  |
| Model 1 | Reference | 1.42 (0.94,2.16) | 1.93 (1.29,2.88) | 2.95 (1.99,4.37) | 1.45 (1.31,1.61) | <0.0001 |  |
| Model 2 | Reference | 1.42 (0.94,2.16) | 1.91 (1.27,2.86) | 2.83 (1.90,4.21) | 1.42 (1.27,1.57) | <0.0001 |  |
| 40-49 years | | | | | | |  |
| Event/Total | 141/2964 | 190/2613 | 278/2777 | 454/3167 |  |  |  |
| Incidence rate | 7.95 | 14.46 | 17.12 | 25.11 |  |  |  |
| Model 1 | Reference | 1.34 (1.07,1.66) | 1.67 (1.36,2.06) | 2.14 (1.75,2.61) | 1.26 (1.20,1.34) | <0.0001 |  |
| Model 2 | Reference | 1.31 (1.05,1.63) | 1.59 (1.29,1.96) | 2.02 (1.65,2.47) | 1.25 (1.18,1.32) | <0.0001 |  |
| 50-59 years | | | | | | |  |
| Event/Total | 190/3365 | 288/3445 | 377/3449 | 538/3664 |  |  |  |
| Incidence rate | 10.03 | 15.25 | 20.19 | 27.53 |  |  |  |
| Model 1 | Reference | 1.37 (1.14,1.64) | 1.70 (1.43,2.03) | 2.16(1.82,2.56) | 1.31 (1.24,1.38) | <0.0001 |  |
| Model 2 | Reference | 1.35 (1.13,1.63) | 1.69 (1.41,2.02) | 2.06(1.74,2.45) | 1.28 (1.22,1.35) | <0.0001 |  |
| 60-69 years | | | | | | | |
| Event/Total | 114/1819 | 187/2094 | 247/2039 | 300/1706 |  |  | |
| Incidence rate | 11.17 | 16.34 | 22.63 | 33.98 |  |  | |
| Model 1 | Reference | 1.31 (1.04,1.66) | 1.70 (1.36,2.14) | 2.37(1.90,2.96) | 1.37 (1.28,1.48) | <0.0001 | |
| Model 2 | Reference | 1.31 (1.03,1.65) | 1.71 (1.36,2.14) | 2.34 (1.87,2.93) | 1.37 (1.27,1.47) | <0.0001 | |
| ≥70 years | | | | | | | |
| Event/Total | 56/963 | 80/984 | 101/917 | 93/595 |  |  | |
| Incidence rate | 11.25 | 16.39 | 22.43 | 33.06 |  |  | |
| Model 1 | Reference | 1.31 (0.93,1.84) | 1.61 (1.15,2.24) | 2.11 (1.50,2.98) | 1.37 (1.20,1.56) | <0.0001 | |
| Model 2 | Reference | 1.31 (0.93,1.84) | 1.60 (1.15,2.24) | 2.08 (1.47,2.94) | 1.36 (1.19,1.55) | <0.0001 | |
| *P-*INTm: CumAIP quartiles × age groups: 0.8789. | | | | | | | |

CumAIP Quartile 1: CumAIP<-0.238378; Q2: -0.2383748≤CumAIP<-0.069924; Q3: -0.069924≤CumAIP<0.0993191; Q4: CumAIP≥0.0993191.

Model 1: adjusted for age (continuous), sex, education, smoking status, drinking status, physical exercise, family history of diabetes, BMI (continuous), antihypertensives (yes or no), and lipid-lowering drugs (yes or no).

Model 2: further adjusted for TC (continuous), hypertension degree (categorical), eGFR (categorical), log(hsCRP, limited to entire cohort).

**^*^**Per SD: hazard ratio per SD change in CumAIP (0.2658).

Abbreviations: CumAIP, cumulative atherogenic index of plasma; CumCRP, cumulative high-sensitivity C-reactive protein; BMI, body mass index; FBG, fasting blood glucose; eGFR, estimated glomerular filtration rate; INTm: multiplicative interaction; TC, total cholesterol.

# Table S3. Age-associated risk of CumCRP thresholds (1,3 mg/L) for type 2 diabetes

|  | **CumCRP, AHRs (95% CIs)** | | | **Per SD** | | ***P*-trend** |
| --- | --- | --- | --- | --- | --- | --- |
|  | **CumCRP < 1 mg/L** | **1 ≤ CumCRP < 3 mg/L** | **CumCRP ≥ 3 mg/L** |  |  |  |
| Event/Total | 860/13882 | 1900/18404 | 1227/10639 |  | |  |
| Incidence rate | 10.83 | 18.34 | 21.33 |  | |  |
| Model 1 | Reference | 1.43 (1.32,1.55) | 1.47 (1.34,1.61) | 1.16 (1.12,1.20) | | <0.0001 |
| Model 2 | Reference | 1.36 (1.25,1.48) | 1.38 (1.26,1.51) | 1.14 (1.10,1.18) | | <0.0001 |
| <40 years | | | | |  |  |
| Event/Total | 68/2443 | 189/2826 | 96/1095 |  | |  |
| Incidence rate | 4.57 | 10.99 | 14.71 |  | |  |
| Model 1 | Reference | 1.89 (1.42,2.50) | 2.16 (1.56,3.00) | 1.35 (1.21,1.51) | | <0.0001 |
| Model 2 | Reference | 1.82 (1.37,2.42) | 2.04 (1.47,2.83) | 1.32 (1.18,1.48) | | <0.0001 |
| 40-50 years | | | | |  |  |
| Event/Total | 294/4461 | 493/4812 | 276/2248 |  | |  |
| Incidence rate | 11.14 | 17.50 | 21.67 |  | |  |
| Model 1 | Reference | 1.31 (1.13,1.52) | 1.48 (1.25,1.75) | 1.17 (1.10,1.24) | | <0.0001 |
| Model 2 | Reference | 1.25 (1.08,1.45) | 1.41 (1.19,1.67) | 1.16 (1.09,1.23) | | <0.0001 |
| 50-60 years | | | | |  |  |
| Event/Total | 296/4277 | 664/6051 | 433/3595 |  | |  |
| Incidence rate | 12.66 | 20.01 | 22.24 |  | |  |
| Model 1 | Reference | 1.44 (1.25,1.65) | 1.50 (1.29,1.75) | 1.17 (1.11,1.23) | | <0.0001 |
| Model 2 | Reference | 1.39 (1.21,1.60) | 1.44 (1.24,1.68) | 1.15 (1.09,1.22) | | <0.0001 |
| 60-70 years |  |  |  |  | |  |
| Event/Total | 151/1912 | 397/3347 | 300/2399 |  | |  |
| Incidence rate | 14.18 | 21.78 | 23.96 |  | |  |
| Model 1 | Reference | 1.39 (1.15,1.69) | 1.47 (1.20,1.79) | 1.15 (1.07,1.23) | | 0.0005 |
| Model 2 | Reference | 1.32 (1.09,1.60) | 1.39 (1.13,1.69) | 1.13 (1.05,1.21) | | 0.0030 |
| ≥70 years |  |  |  |  | |  |
| Event/Total | 51/789 | 157/1368 | 122/1302 |  | |  |
| Incidence rate | 12.43 | 23.10 | 19.45 |  | |  |
| Model 1 | Reference | 1.60 (1.16,2.20) | 1.27 (0.91,1.77) | 1.05 (0.94,1.18) | | 0.4735 |
| Model 2 | Reference | 1.53 (1.11,2.10) | 1.18 (0.85,1.65) | 1.03 (0.92,1.16) | | 0.7904 |
| *P*-INTm: CumCRP Cut-offs (1, 3 mg/L) × age groups= 0.0784; *log*CumCRP × age groups=0.0027 | | | | | | |

Model 1: adjusted for age (continuous), sex, education, smoking status, drinking status, physical exercise, family history of diabetes, BMI (continuous), antihypertensives (yes or no), and lipid-lowering drugs (yes or no).

Model 2: Model 1 plus log (TG/HDL-C) (continuous), hypertension degree (categorical), eGFR (categorical), antihypertensives (yes or no), and lipid-lowering drugs (yes or no).

Per SD, average hazard ratio per SD (0.4275) changes in LogCumCRP.

Abbreviations: AHRs: average hazard ratios; CI: confidence intervals; CumAIP, cumulative atherogenic index of plasma; CumCRP, cumulative high-sensitivity C-reactive protein; BMI, body mass index; FBG, fasting blood glucose; eGFR, estimated glomerular filtration rate; INTm: multiplicative interaction; TC, total cholesterol.

# Table S4. CumAIP-associated type 2 diabetes risks across different CumCRP strata (<1,1-3,≥3 mg/L)

|  | **CumAIP, HRs (95% CIs)** | | | | **Per SD^*^** | ***P* for trend** |  |
| --- | --- | --- | --- | --- | --- | --- | --- |
|  | **Quartile1** | **Quartile2** | **Quartile3** | **Quartile4** |  |  |  |
| CumCRP<1 mg/L | | | | | | |  |
| Incidence rate | 5.75 | 8.38 | 13.18 | 20.30 |  |  |  |
| Model 1 | Reference | 1.23 (0.99,1.54) | 1.79 (1.46,2.21) | 2.58 (2.10,3.18) | 1.44 (1.35,1.54) | <0.0001 |  |
| Model 2 | Reference | 1.20 (0.96,1.50) | 1.67 (1.35,2.06) | 2.38 (1.93,2.93) | 1.40 (1.31,1.50) | <0.0001 |  |
| 1≤CumCRP<3 mg/L | | | | | | |  |
| Incidence rate | 10.52 | 15.18 | 19.45 | 26.69 |  |  |  |
| Model 1 | Reference | 1.30 (1.11,1.53) | 1.57 (1.35,1.84) | 2.09 (1.80,2.43) | 1.30 (1.24,1.36) | <0.0001 |  |
| Model 2 | Reference | 1.29 (1.10,1.51) | 1.54 (1.32,1.80) | 2.03 (1.74,2.36) | 1.28 (1.22,1.34) | <0.0001 |  |
| CumCRP≥3 mg/L | | | | | | |  |
| Incidence rate | 11.33 | 17.34 | 22.93 | 31.20 |  |  |  |
| Model 1 | Reference | 1.38 (1.13,1.70) | 1.70 (1.40,2.07) | 2.17 (1.80,2.62) | 1.28 (1.21,1.35) | <0.0001 |  |
| Model 2 | Reference | 1.37 (1.11,1.68) | 1.67 (1.37,2.04) | 2.10 (1.74,2.54) | 1.27 (1.20,1.34) | <0.0001 |  |
| *P* -INTm: CumAIP quartiles × CumCRP (<1,1-3,≥3 mg/L): 0.1065. CumAIP quartiles * *log*CumCRP=0.0443. | | | | | | | |

Model 1: adjusted for age (continuous), sex, education, smoking status, drinking status, physical exercise, family history of diabetes, BMI (continuous), antihypertensives (yes or no), and lipid-lowering drugs (yes or no).

Model 2: further adjusted for TC (continuous), hypertension degree (categorical), eGFR (categorical), antihypertensives (yes or no), and lipid-lowering drugs (yes or no).

**^*^**Per SD: hazard ratio per SD change in CumAIP (0.2658).

Abbreviations: CumAIP, cumulative atherogenic index of plasma; CumCRP, cumulative high-sensitivity C-reactive protein; BMI, body mass index; FBG, fasting blood glucose; eGFR, estimated glomerular filtration rate; INTm: multiplicative interaction; TC, total cholesterol.

# Table S5. Age-associated risk of co-exposure to CumAIP and CumCRP for incident type 2 diabetes

|  | **Combination of CumCRP and CumAIP, HRs (95% CIs)** | | | | | |  |
| --- | --- | --- | --- | --- | --- | --- | --- |
|  | **CumAIP<-0.0699& CumCRP<1 mg/L** | **CumAIP<-0.0699 &**  **1≤ CumCRP<3 mg/L** | **CumAIP<-0.0699 & CumCRP≥3 mg/L** | **CumAIP≥-0.0699 & CumCRP<1 mg/L** | **CumAIP≥-0.0699 & 1≤CumCRP<3 mg/L** | **CumAIP≥-0.0699 & CumCRP≥3 mg/L** |  |
| **Entire cohort** | | | | | | |  |
| Unadjusted model | Reference | 1.88 (1.64,2.15) | 2.05 (1.77,2.38) | 2.34 (2.04,2.68) | 3.31 (2.93,3.74) | 3.92 (2.45,4.46) |  |
| Model 1 | Reference | 1.63 (1.42,1.87) | 1.60 (1.38,1.86) | 1.98 (1.72,2.28) | 2.48 (2.19,2.81) | 2.62 (2.30,3.00) |  |
| Model 2 | Reference | 1.61 (1.40,1.84) | 1.59 (1.37,1.85) | 1.91 (1.66,2.20) | 2.38 (2.10,2.70) | 2.53 (2.21,2.89) |  |
| *P*-INTm: CumAIP median × CumCRP cut-points (1,3 mg/L) = 0.0126 | | | | | | |  |
| **<40 years** | | | | | | |  |
| Unadjusted model | Reference | 4.19 (2.37,7.41) | 5.23 (2.76,9.91) | 5.17 (2.91,9.17) | 8.37 (4.91,14.28) | 11.49 (6.58,20.05) |  |
| Model 1 | Reference | 3.62 (2.04,6.43) | 4.00 (2.10,7.64) | 4.02 (2.24,7.22) | 5.60 (3.22,9.75) | 6.67 (3.71, 11.98) |  |
| Model 2 | Reference | 3.60 (2.03,6.39) | 3.97 (2.08,7.59) | 3.75 (2.08,6.77) | 5.33 (3.06,9.31) | 6.26 (3.47,11.28) |  |
| *P*-INTm: CumAIP median × CumCRP cut-points (1, 3 mg/L) = 0.0178 | | | | | | |  |
| **40-49 years** | | | | | | |  |
| Unadjusted model | Reference | 1.55 (1.21,1.99) | 1.97 (1.48,2.62) | 2.19 (1.73,2.77) | 3.01 (2.43,3.72) | 3.74 (2.96,4.72) |  |
| Model 1 | Reference | 1.38 (1.08,1.78) | 1.60 (1.20,2.14) | 1.78 (1.40,2.26) | 2.13 (1.71,2.66) | 2.41 (1.89,3.07) |  |
| Model 2 | Reference | 1.36 (1.05,1.74) | 1.56 (1.17,2.08) | 1.64 (1.29,2.09) | 1.99 (1.59,2.49) | 2.26 (1.77,2.89) |  |
| *P*-INTm: CumAIP median × CumCRP cut-points (1, 3 mg/L) = 0.7255 | | | | | | |  |
| **50-59 years** | | | | | | | |
| Unadjusted model | Reference | 1.80 (1.43,2.26) | 1.97 (1.54,2.53) | 2.31 (1.82,2.92) | 3.08 (2.49,3.80) | 3.35 (2.68,4.18) |  |
| Model 1 | Reference | 1.68 (1.33,2.11) | 1.78 (1.39,2.30) | 2.01 (1.58,2.55) | 2.51 (2.02,3.11) | 2.60 (2.07,3.27) |  |
| Model 2 | Reference | 1.66 (1.32,2.09) | 1.80 (1.40,2.32) | 1.94 (1.52,2.46) | 2.43 (1.96,3.01) | 2.51 (2.00,3.16) |  |
| *P*-INTm: CumAIP median × CumCRP cut-points (1, 3 mg/L) = 0.0861 | | | | | | |  |
| **60-69 years** | | | | | | |  |
| Unadjusted model | Reference | 1.56 (1.16,2.08) | 1.43 (1.04,1.97) | 1.97 (1.43,2.72) | 2.71 (2.06,3.56) | 3.14 (2.37,4.14) |  |
| Model 1 | Reference | 1.44 (1.07,1.93) | 1.30 (0.94,1.80) | 1.68 (1.22,2.33) | 2.21 (1.68,2.92) | 2.48 (1.86,3.30) |  |
| Model 2 | Reference | 1.41 (1.05,1.89) | 1.29 (0.93,1.78) | 1.66 (1.20,2.30) | 2.14 (1.62,2.83) | 2.48 (1.86,3.30) |  |
| *P*-INTm: CumAIP median × CumCRP cut-points (1, 3 mg/L) = 0.3474 | | | | | | |  |
| **≥70 years** | | | | | | |  |
| Unadjusted model | Reference | 2.05 (1.27,3.29) | 1.55 (0.94,2.54) | 2.24 (1.29,3.89) | 3.30 (2.09,5.21) | 3.10 (1.94,4.95) |  |
| Model 1 | Reference | 1.84 (1.14,2.96) | 1.30 (0.79,2.15) | 1.80 (1.03,3.14) | 2.43 (1.53,3.87) | 2.16 (1.34,3.49) |  |
| Model 2 | Reference | 1.80 (1.11,2.90) | 1.27 (0.77,2.10) | 1.73 (0.99,3.02) | 2.37 (1.48,3.78) | 2.10 (1.29,3.40) |  |
| *P*-INTm: CumAIP median × CumCRP cut-points (1, 3 mg/L) = 0.5683 | | | | | | |  |

*P*-INTm: Co- exposure subgroups and age-groups: 0.0193 (Model 2).

Model 1: adjusted for age, sex, education, smoking status, drinking status, physical exercise, family history of diabetes, BMI, antihypertensives (yes or no), and lipid-lowering agents (yes or no).

Model 2: model 1 + TC, hypertension degree, eGFR.

Abbreviations: CumAIP, cumulative atherogenic index of plasma; CumCRP, cumulative high-sensitivity C-reactive protein; CI, confidence interval; eGFR, estimated glomerular filtration rate; FBG, fasting blood glucose; HR, hazard ratio; TC, total cholesterol; INTm: multiplicative interaction.

# Table S6. Age-associated risk of co-exposure to CumCRP and CumAIP for type 2 diabetes (reference group: CumAIP<-0.0699 & 1≤ CumCRP <3mg/L)

| **Combination of CumCRP and CumAIP, HRs (95% CIs)** | | | | | | |
| --- | --- | --- | --- | --- | --- | --- |
|  | **CumAIP<-0.0699 & 1≤CumCRP<3 mg/L** | **CumAIP<-0.0699 & CumCRP<1 mg/L** | **CumAIP<-0.0699 & CumCRP≥3 mg/L** | **CumAIP≥-0.0699 & CumCRP<1 mg/L** | **CumAIP≥-0.0699 & 1≤CumCRP<3 mg/L** | **CumAIP≥-0.0699 & CumCRP≥3 mg/L** |
| <40 years | | | | | | |
| Model | Reference | 0.28 (0.16,0.49) | 1.10 (0.69,1.77) | 1.04 (0.71,1.54) | 1.48 (1.07,2.06) | 1.74 (1.19,2.53) |
| 40-49 years | | | | | | |
| Model | Reference | 0.74 (0.57,0.95) | 1.15 (0.87,1.52) | 1.21 (0.97,1.51) | 1.47 (1.20,1.80) | 1.67 (1.33,2.09) |
| 50-59 years | | | | | | |
| Model | Reference | 0.60 (0.48,0.76) | 1.08 (0.88,1.34) | 1.16 (0.96,1.41) | 1.46 (1.24,1.72) | 1.51 (1.26,1.81) |
| 60-69 years | | | | | | |
| Model | Reference | 0.71 (0.53,0.95) | 0.91 (0.70,1.19) | 1.18 (0.90,1.54) | 1.52 (1.23,1.86) | 1.76 (1.42,2.18) |
| ≥70 years | | | | | | |
| Model | Reference | 0.56 (0.35,0.90) | 0.71 (0.49,1.03) | 0.97 (0.62,1.51) | 1.32 (0.95,1.83) | 1.17 (0.83,1.65) |

Model: adjusted for age (continuous), sex, education, smoking status, drinking status, physical exercise, family history of diabetes, BMI (continuous), eGFR (categorical), TC (continuous), hypertension degree (categorical), antihypertensives (yes or no), and lipid-lowering drugs (yes or no).

# Table S7. Age-associated risk of co-exposure to CumCRP and CumAIP for type 2 diabetes (reference group: CumAIP≥-0.0699 & CumCRP≥3 mg/L)

| **Combination of CumCRP and CumAIP, HRs (95% CIs)** | | | | | | |
| --- | --- | --- | --- | --- | --- | --- |
|  | **CumAIP≥-0.0699 & CumCRP≥3 mg/L** | **CumAIP<-0.0699 & CumCRP<1 mg/L** | **CumAIP<-0.0699 & 1≤CumCRP<3 mg/L** | **CumAIP<-0.0699 & CumCRP≥3 mg/L** | **CumAIP≥-0.0699 & CumCRP<1 mg/L** | **CumAIP≥-0.0699 & 1≤CumCRP<3 mg/L** |
| <40 years | | | | | | |
| Model | Reference | 0.16 (0.09,0.29) | 0.58 (0.40,0.84) | 0.64 (0.40,1.02) | 0.60 (0.41,0.87) | 0.85 (0.63,1.15) |
| 40-49 years | | | | | | |
| Model | Reference | 0.44 (0.35,0.57) | 0.60 (0.48,0.75) | 0.69 (0.53,0.90) | 0.73 (0.59,0.89) | 0.88 (0.74,1.05) |
| 50-59 years | | | | | | |
| Model | Reference | 0.40 (0.32,0.50) | 0.66 (0.55,0.79) | 0.72 (0.59,0.88) | 0.77 (0.64,0.93) | 0.97 (0.83,1.12) |
| 60-69 years | | | | | | |
| Model | Reference | 0.40 (0.30,0.54) | 0.57 (0.46,0.71) | 0.52 (0.41,0.67) | 0.67 (0.52,0.87) | 0.86 (0.72,1.04) |
| ≥70 years | | | | | | |
| Model | Reference | 0.48 (0.30,0.78) | 0.86 (0.61,1.21) | 0.61 (0.42,0.88) | 0.83 (0.53,1.28) | 1.13 (0.83,1.54) |

Model: adjusted for age (continuous), sex, education, smoking status, drinking status, physical exercise, family history of diabetes, BMI (continuous), eGFR (categorical), TC (continuous), hypertension degree (categorical), antihypertensives (yes or no), and lipid-lowering drugs (yes or no).

# Table S8. Age-associated risk of co-exposure to CumCRP and CumAIP for type 2 diabetes (reference group: CumAIP≥-0.0699 & 1≤CumCRP<3 mg/L)

| **Combination of CumCRP and CumAIP, HRs (95% CIs)** | | | | | | |
| --- | --- | --- | --- | --- | --- | --- |
|  | **CumAIP≥-0.0699 & 1≤CumCRP<3 mg/L** | **CumAIP<-0.0699 & CumCRP<1 mg/L** | **CumAIP<-0.0699 & 1≤ CumCRP<3 mg/L** | **CumAIP<-0.0699 & CumCRP≥3 mg/L** | **CumAIP≥-0.0699 & CumCRP<1 mg/L** | **CumAIP≥-0.0699 & CumCRP≥3 mg/L** |
| <40 years | | | | | | |
| Model | Reference | 0.19 (0.11,0.33) | 0.68 (0.49,0.94) | 0.75 (0.48,1.15) | 0.70 (0.51,0.97) | 1.17 (0.87,1.58) |
| 40-49 years | | | | | | |
| Model | Reference | 0.50 (0.40,0.63) | 0.68 (0.56,0.83) | 0.78 (0.61,1.00) | 0.82 (0.69,0.99) | 1.14 (0.95,1.36) |
| 50-59 years | | | | | | |
| Model | Reference | 0.41 (0.33,0.51) | 0.69 (0.58,0.81) | 0.74 (0.61,0.90) | 0.80 (0.67,0.95) | 1.04 (0.89,1.20) |
| 60-69 years | | | | | | |
| Model | Reference | 0.47 (0.35,0.62) | 0.66 (0.54,0.81) | 0.60 (0.47,0.77) | 0.78 (0.61,0.99) | 1.16 (0.96,1.40) |
| ≥70 years | | | | | | |
| Model | Reference | 0.42 (0.27,0.67) | 0.76 (0.55,1.05) | 0.54 (0.38,0.77) | 0.73 (0.48,1.12) | 0.88 (0.65,1.21) |

Model: adjusted for age (continuous), sex, education, smoking status, drinking status, physical exercise, family history of diabetes, BMI (continuous), eGFR (categorical), TC (continuous), hypertension degree (categorical), antihypertensives (yes or no), and lipid-lowering drugs (yes or no).

# Table S9. Additive effect of elevated chronic inflammation and dyslypidemia on developing type 2 diabetes

| **Main effects ‒ hazard ratios** | **Model 1** | **Model 2** |
| --- | --- | --- |
| Entire cohort | | |
| CumCRP≥3 mg/L | 1.30 (1.16‒1.47) | 1.29 (1.15‒1.45) |
| CumAIP≥median | 1.99 (1.84‒2.16) | 1.89 (1.75‒2.05) |
| Joint effect | 2.49 (2.27‒2.74) | 2.36 (2.14‒2.09) |
| RERI | 0.20 (-0.04‒0.44) | 0.17 (-0.06‒0.40) |
| AP | 0.08 (-0.01‒0.17) | 0.07 (-0.02‒0.17) |
| Age <45 years | | |
| CumCRP≥3 mg/L | 1.56 (1.12‒2.17) | 1.50 (1.08‒2.09) |
| CumAIP≥median | 1.93 (1.59‒2.33) | 1.72 (1.41‒2.08) |
| Joint effect | 3.49 (2.77‒4.39) | 3.02 (2.39‒3.82) |
| RERI | 1.00 (0.23‒1.77) | 0.80 (0.10‒1.50) |
| AP | 0.29 (0.09‒0.48) | 0.27 (0.06‒0.47) |
| 45<age≤65 years | | |
| CumCRP≥3 mg/L | 1.42 (1.23‒1.64) | 1.42 (1.23‒1.64) |
| CumAIP≥median | 2.01 (1.82‒2.21) | 1.90 (1.73‒2.10) |
| Joint effect | 2.36 (2.10‒2.65) | 2.25 (2.00‒2.53) |
| RERI | -0.07 (-0.36‒0.23) | -0.07 (-0.36‒0.21) |
| AP | -0.03 (-0.16‒0.10) | -0.03 (-0.16‒0.10) |
| Age≥65 years | | |
| CumCRP≥3 mg/L | 0.90 (0.68‒1.18) | 0.89 (0.67‒1.17) |
| CumAIP≥median | 1.79 (1.45‒2.20) | 1.76 (1.43‒2.16) |
| Joint effect | 2.15 (1.72‒2.70) | 2.12 (1.69‒2.66) |
| RERI | 0.47 (0.001‒0.94) | 0.48 (0.01‒0.94) |
| AP | 0.22 (0.02‒0.42) | 0.23 (0.02‒0.43) |

Model 1: adjusted for age, sex, education, smoking status, drinking status, physical exercise, family history of diabetes, antihypertensives (yes or no), and lipid-lowering agents (yes or no).

Model 2: model 1 + TC, hypertension degree, eGFR.

Baseline category was defined as CumCRP<3 mg/L and CumAIP<median (-0.0699).

Abbreviations: RERI, relative excess risk due to interaction; AP, attributable proportion due to interaction; others are as eTable 2.

# Table S10. Sensitivity analysis of age-associated risks of co-exposure of CumCRP and CumAIP for type 2 diabetes by additional adjustment for baseline fatty liver degree (3987/42925)

|  | **Combination of CumCRP and CumAIP, HRs (95% CIs)** | | | | | |
| --- | --- | --- | --- | --- | --- | --- |
|  | **CumCRP<1 mg/L & CumAIP<-0.0699** | **1≤CumCRP<3 mg/L & CumAIP<-0.0699** | **CumCRP≥3 mg/L & CumAIP<-0.0699** | **CumCRP<1 mg/L & CumAIP≥-0.0699** | **1≤CumCRP<3 mg/L & CumAIP≥-0.0699** | **CumCRP≥3 mg/L & CumAIP≥-0.0699** |
| **Entire cohort** | | | | | | |
| Model | Reference | 1.55 (1.35,1.77) | 1.51 (1.30,1.75) | 1.77 (1.54,2.04) | 2.06 (1.81,2.34) | 2.12 (1.85,2.43) |
| *P*-INTm: CumAIP median × CumCRP thresholds (1, 3 mg/L) = 0.0034 | | | | | | |
| **<40 years** | | | | | | |
| Model | Reference | 3.55 (2.00,6.30) | 3.71 (1.94,7.11) | 3.54 (1.96,6.41) | 4.60 (2.62,8.08) | 5.10 (2.81,9.28) |
| *P*-INTm: CumAIP median × CumCRP thresholds (1, 3 mg/L) = 0.0089 | | | | | | |
| **40-49 years** | | | | | | |
| Model | Reference | 1.31 (1.02,1.69) | 1.46 (1.09,1.95) | 1.55 (1.21,1.97) | 1.73 (1.38,2.18) | 1.81 (1.41,2.33) |
| *P*-INTm: CumAIP median × CumCRP thresholds (1, 3 mg/L) = 0.4400 | | | | | | |
| **50-59 years** | | | | | | |
| Model | Reference | 1.60 (1.27,2.02) | 1.70 (1.32,2.20) | 1.81 (1.42,2.30) | 2.12 (1.71,2.64) | 2.14 (1.69,2.69) |
| *P*-INTm: CumAIP median × CumCRP thresholds (1, 3 mg/L) = 0.0493 | | | | | | |
| **60-69 years** | | | | | | |
| Model | Reference | 1.38 (1.03,1.85) | 1.24 (0.90,1.72) | 1.55 (1.12,2.15) | 1.87(1.441,2.48) | 2.17 (1.63,2.91) |
| *P*-INTm: CumAIP median × CumCRP thresholds (1, 3 mg/L) = 0.3005 | | | | | | |
| **≥70 years** | | | | | | |
| Model | Reference | 1.75 (1.08,2.83) | 1.26 (0.76,2.08) | 1.58 (0.90,2.76) | 2.13 (1.33,3.41) | 1.86 (1.14,3.03) |
| *P*-INTm: CumAIP median × CumCRP thresholds (1, 3 mg/L) = 0.6336 | | | | | | |

*P* -INTm: Co-exposure × age groups = 0.0894.

Model: adjusted for age (continuous), sex, education, smoking status, drinking status, physical exercise, family history of diabetes, BMI (continuous), eGFR (categorical), TC (continuous), hypertension degree (categorical), antihypertensives (yes or no), lipid-lowering drugs (yes or no), and fatty liver degree (mild, moderate, severe).

Abbreviations as Table S2.

# Table S11. Reverse analysis of age-associated risk of co-exposure to CumCRP and CumAIP for type 2 diabetes by excluding diabetes onset within the first follow-up visit (2492/41430)

| **Combination of CumCRP and CumAIP, HRs (95% CIs)** | | | | | | |
| --- | --- | --- | --- | --- | --- | --- |
|  | **CumCRP<1 mg/L & CumAIP<-0.0699** | **1≤CumCRP<3 mg/L & CumAIP<-0.0699** | **CumCRP≥3 mg/L & CumAIP<-0.0699** | **CumCRP<1 mg/L & CumAIP≥-0.0699** | **1≤CumCRP<3 mg/L & CumAIP≥-0.0699** | **CumCRP≥3 mg/L & CumAIP≥-0.0699** |
| Entire cohort | | | | | | |
| Model | Reference | 1.80 (1.51,2.14) | 1.41 (1.15,1.73) | 2.44 (2.05,2.92) | 2.81 (2.39,3.31) | 2.56 (2.15,3.06) |
| <40 years | | | | | | |
| Model | Reference | 5.22 (2.55,10.67) | 4.46 (1.96,10.17) | 5.89 (2.83,12.26) | 7.76 (3.83,15.71) | 8.03 (3.81,16.93) |
| 40-49 years | | | | | | |
| Model | Reference | 1.52 (1.11,2.07) | 1.29 (0.87,1.90) | 1.92 (1.43,2.59) | 2.39 (1.81,3.15) | 2.57 (1.89,3.49) |
| 50-59 years | | | | | | |
| Model | Reference | 1.67 (1.23,2.27) | 1.49 (1.05,2.11) | 2.53 (1.86,3.44) | 2.66 (2.00,3.54) | 2.40 (1.77,3.26) |
| 60-69 years | | | | | | |
| Model | Reference | 1.67 (1.15,2.43) | 1.25 (0.82,1.92) | 2.24 (1.49,3.34) | 2.50 (1.75,3.59) | 2.18 (1.49,3.20) |
| ≥70 years | | | | | | |
| Model | Reference | 2.30 (1.09,4.84) | 1.24 (0.55,2.78) | 2.86 (1.27,6.42) | 3.86 (1.88,7.90) | 2.45 (1.15,5.21) |

*P*-INTm in the entire cohort: CumAIP median × CumCRP thresholds (1, 3, mg/L) =0.0002; Co-exposure × age groups = 0.0189.

Model: adjusted for age (continuous), sex, education, smoking status, drinking status, physical exercise, family history of diabetes, BMI (continuous), eGFR (categorical), TC (continuous), hypertension degree (categorical), antihypertensives (yes or no), and lipid-lowering drugs (yes or no).

Abbreviations as Table S2.

# Table S12. Sensitivity analysis of age-associated risks of co-exposure of CumCRP and CumAIP for type 2 diabetes by excluding baseline CVD (3719/40713)

| **Combination of CumCRP and CumAIP, HRs (95% CIs)** | | | | | | |
| --- | --- | --- | --- | --- | --- | --- |
|  | **CumCRP<1 mg/L & CumAIP<-0.0699** | **1≤CumCRP<3 mg/L & CumAIP<-0.0699** | **CumCRP≥3 mg/L & CumAIP<-0.0699** | **CumCRP<1 mg/L & CumAIP≥-0.0699** | **1≤CumCRP<3 mg/L & CumAIP≥-0.0699** | **CumCRP≥3 mg/L & CumAIP≥-0.0699** |
| Entire cohort | | | | | | |
| Model | Reference | 1.65 (1.43,1.90) | 1.62 (1.38,1.89) | 1.96 (1.70,2.27) | 2.49 (2.19,2.84) | 2.71 (2.35,3.11) |
| <40 years | | | | | | |
| Model | Reference | 3.60 (2.03,6.39) | 3.97 (2.08,7.57) | 3.77 (2.09,6.80) | 5.34 (3.06, 9.31) | 6.28 (3.48,11.33) |
| 40-49 years | | | | | | |
| Model | Reference | 1.38 (1.07,1.78) | 1.58 (1.18,2.12) | 1.69 (1.32,2.15) | 2.06 (1.64,2.58) | 2.37 (1.85,3.03) |
| 50-59 years | | | | | | |
| Model | Reference | 1.71 (1.35,2.16) | 1.75 (1.34,2.27) | 1.94 (1.51,2.48) | 2.46 (1.97,3.08) | 2.60 (2.05,3.29) |
| 60-69 years | | | | | | |
| Model | Reference | 1.40 (1.02,1.92) | 1.35 (0.96,1.90) | 1.66 (1.18,2.36) | 2.28 (1.70,3.08) | 2.71 (1.99,3.68) |
| ≥70 years | | | | | | |
| Model | Reference | 2.15 (1.24,3.71) | 1.47 (0.83,2.60) | 1.95 (1.03,3.69) | 2.96 (1.73, 5.07) | 2.80 (1.61,4.84) |

*P-*INTm in the entire cohort: CumAIP median × CumCRP thresholds (1, 3, mg/L) = 0.0133; Co-exposure × age groups = 0.0716.

Model: adjusted for age (continuous), sex, education, smoking status, drinking status, physical exercise, family history of diabetes, BMI (continuous), eGFR (categorical), TC (continuous), hypertension degree (categorical), antihypertensives (yes or no), and lipid-lowering drugs (yes or no).

Abbreviations as Table S2.

# Table S13. Sensitivity analysis of age-associated risks of co-exposure of CumCRP and CumAIP for type 2 diabetes by excluding impaired fasting glucose in exposure period (2061/35287)

| **Combination of CumCRP and CumAIP, HRs (95% CIs)** | | | | | | |
| --- | --- | --- | --- | --- | --- | --- |
|  | **CumCRP<1 mg/L & CumAIP<-0.0699** | **1≤CumCRP<3 mg/L & CumAIP<-0.0699** | **CumCRP≥3 mg/L & CumAIP<-0.0699** | **CumCRP<1 mg/L & CumAIP≥-0.0699** | **1≤CumCRP<3 mg/L & CumAIP≥-0.0699** | **CumCRP≥3 mg/L & CumAIP≥-0.0699** |
| Entire cohort | | | | | | |
| Model | Reference | 1.77 (1.48,2.12) | 1.61 (1.31,1.98) | 1.84 (1.51,2.23) | 2.42 (2.04,2.88) | 2.68 (2.23,3.22) |
| <40 years | | | | | | |
| Model | Reference | 4.79 (2.32,9.88) | 5.84 (2.65,12.86) | 4.55 (2.14,9.69) | 6.84 (3.35,13.96) | 6.91 (3.23,14.78) |
| 40-49 years | | | | | | |
| Model | Reference | 1.54 (1.12,2.11) | 1.50 (1.04,2.18) | 1.51 (1.10,2.08) | 1.83 (1.37,2.45) | 2.27 (1.65,3.11) |
| 50-59 years | | | | | | |
| Model | Reference | 1.83 (1.30,2.57) | 1.95 (1.35,2.83) | 2.08 (1.45,2.97) | 2.76 (2.00,3.80) | 2.92 (2.09,4.09) |
| 60-69 years | | | | | | |
| Model | Reference | 1.43 (0.96,2.12) | 1.18 (0.75,1.85) | 1.29 (0.81,2.07) | 1.92 (1.31,2.82) | 2.45 (1.65,3.62) |
| ≥70 years | | | | | | |
| Model | Reference | 1.81 (0.95,3.45) | 0.94 (0.46,1.90) | 1.31 (0.58,2.95) | 2.14 (1.12, 4.06) | 1.87 (0.96,3.65) |

*P* for interaction in the entire cohort: CumAIP median × CumCRP thresholds (1, 3, mg/L) = 0.0273; Co-exposure × age groups = 0.0180.

Model: adjusted for age (continuous), sex, education, smoking status, drinking status, physical exercise, family history of diabetes, BMI (continuous), eGFR (categorical), TC (continuous), hypertension degree (categorical), antihypertensives (yes or no), and lipid-lowering drugs (yes or no).

Abbreviations as Table S2.

# Table S14. Sensitivity analysis of age (<40,40-49,50-59,60-69,≥70)-associated risks of co-exposure of CumCRP and CumAIP for type 2 diabetes on raw data (3977/42807)

| **Combination of CumCRP and CumAIP, HRs (95% CIs)** | | | | | | |
| --- | --- | --- | --- | --- | --- | --- |
|  | **CumCRP<1 mg/L & CumAIP<-0.0699** | **1≤CumCRP<3 mg/L & CumAIP<-0.0699** | **CumCRP≥3 mg/L & CumAIP<-0.0699** | **CumCRP<1 mg/L & CumAIP≥-0.0699** | **1≤CumCRP<3 mg/L & CumAIP≥-0.0699** | **CumCRP≥3 mg/L & CumAIP≥-0.0699** |
| Entire cohort | | | | | | |
| Model | Reference | 1.61 (1.41,1.84) | 1.59 (1.37,1.85) | 1.90 (1.65,2.19) | 2.38 (2.10,2.70) | 2.52 (2.20,2.88) |
| <40 years | | | | | | |
| Model | Reference | 3.62 (2.04,6.42) | 3.91 (2.04,7.47) | 3.75 (2.08,6.75) | 5.33 (3.06,9.31) | 6.23 (3.46,11.25) |
| 40-49 years | | | | | | |
| Model | Reference | 1.36 (1.06,1.74) | 1.56 (1.17,2.08) | 1.64 (1.29,2.09) | 1.99 (1.58,2.49) | 2.25 (1.76,2.87) |
| 50-59 years | | | | | | |
| Model | Reference | 1.66 (1.32,2.09) | 1.80 (1.40,2.32) | 1.90 (1.50,2.42) | 2.42 (1.95,3.00) | 2.49 (1.98,3.14) |
| 60-69 years | | | | | | |
| Model | Reference | 1.41 (1.05,1.89) | 1.29 (0.93,2.78) | 1.67 (1.21,2.31) | 2.13 (1.61,2.82) | 2.47 (1.86,3.30) |
| ≥70 years | | | | | | |
| Model | Reference | 1.88 (1.16,3.06) | 1.33 (0.80,2.22) | 1.80 (1.03,3.17) | 2.46 (1.53, 3.95) | 2.18 (1.33,3.56) |

*P* for interaction in the entire cohort: CumAIP median × CumCRP thresholds (1, 3 mg/L) = 0.0131; Co-exposure × age groups = 0.0287.

Model: adjusted for age (continuous), sex, education, smoking status, drinking status, physical exercise, family history of diabetes, BMI (continuous), eGFR (categorical), TC (continuous), hypertension degree (categorical), antihypertensives (yes or no), and lipid-lowering drugs (yes or no).

Abbreviations as Table S2.

# Table S15. Sensitivity analysis of age(<45, 45-64,≥65)-associated risks of co-exposure of CumCRP and CumAIP for type 2 diabetes on raw data (3977/42807)

| **Combination of CumCRP and CumAIP, HRs (95% CIs)** | | | | | | |
| --- | --- | --- | --- | --- | --- | --- |
|  | **CumCRP<1 mg/L & CumAIP<-0.0699** | **1≤CumCRP<3 mg/L & CumAIP<-0.0699** | **CumCRP≥3 mg/L & CumAIP<-0.0699** | **CumCRP<1 mg/L & CumAIP≥-0.0699** | **1≤CumCRP<3 mg/L & CumAIP≥-0.0699** | **CumCRP≥3 mg/L & CumAIP≥-0.0699** |
| <45 years | | | | | | |
| Model | Reference | 1.91 (1.39,2.63) | 1.83 (1.23,2.71) | 1.85 (1.34,2.55) | 2.22 (1.64,3.00) | 3.01 (2.17,4.19) |
| 45-64 years | | | | | | |
| Model | Reference | 1.51 (1.28,1.78) | 1.69 (1.41,2.03) | 1.82 (1.53,2.15) | 2.36 (2.03,2.74) | 2.37 (2.02,2.79) |
| ≥65 years | | | | | | |
| Model | Reference | 1.81 (1.26,2.61) | 1.28 (0.87,1.89) | 2.15 (1.44,3.22) | 2.41 (1.69, 3.45) | 2.69 (1.87,3.86) |

*P* for interaction in the entire cohort: Co-exposure × age groups = 0.0706.

Model: adjusted for age (continuous), sex, education, smoking status, drinking status, physical exercise, family history of diabetes, BMI (continuous), eGFR (categorical), TC (continuous), hypertension degree(categorical), antihypertensives (yes or no), and lipid-lowering drugs (yes or no).

Abbreviations as Table S2.


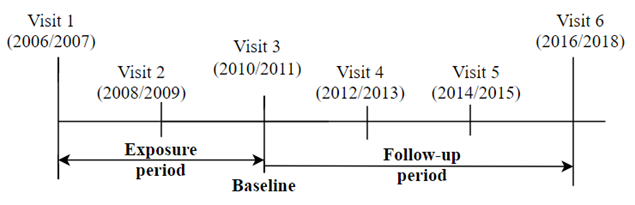


#
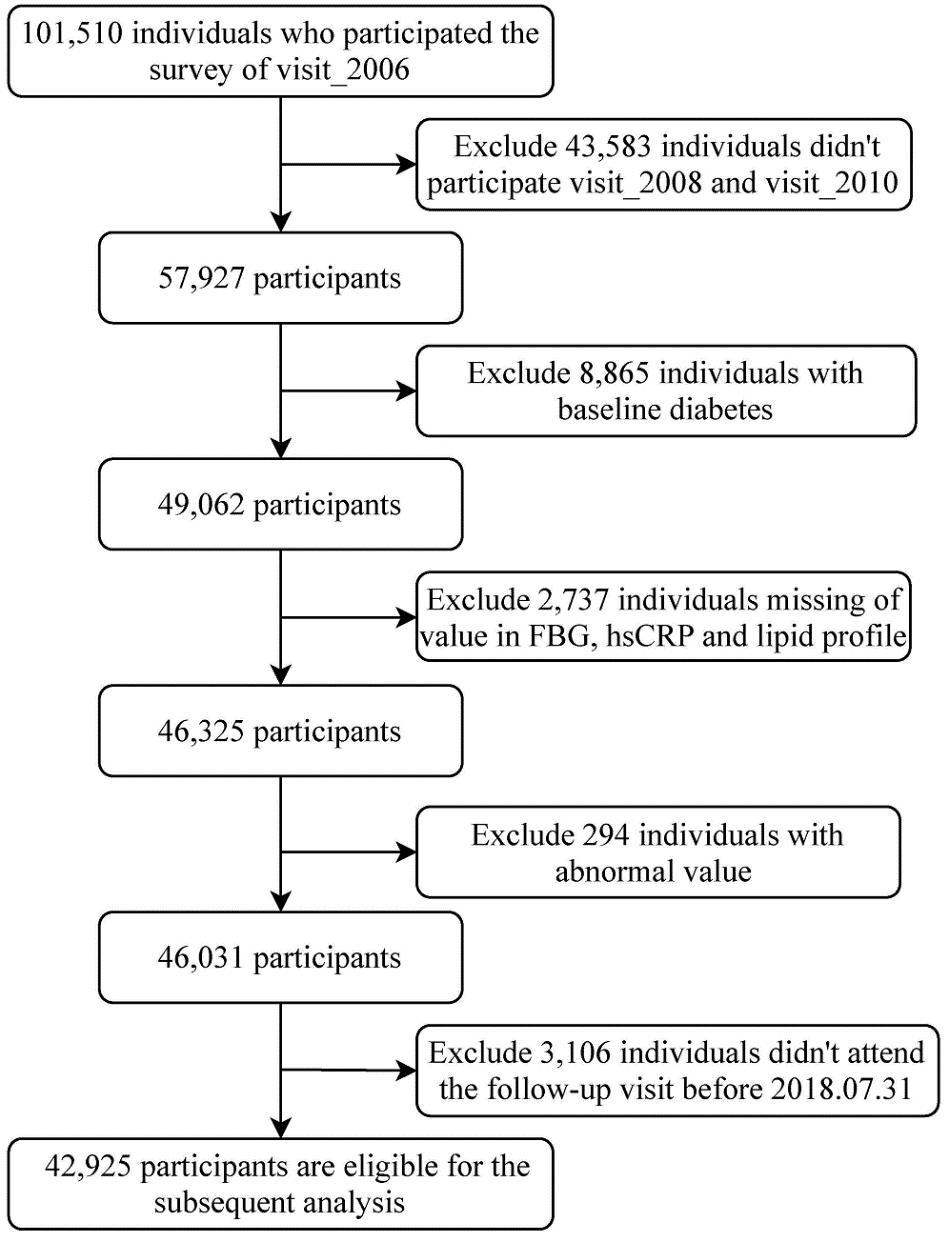
Fig. S1 Strategy of the study design

# Fig. S2 Flowchart of the study participants

#
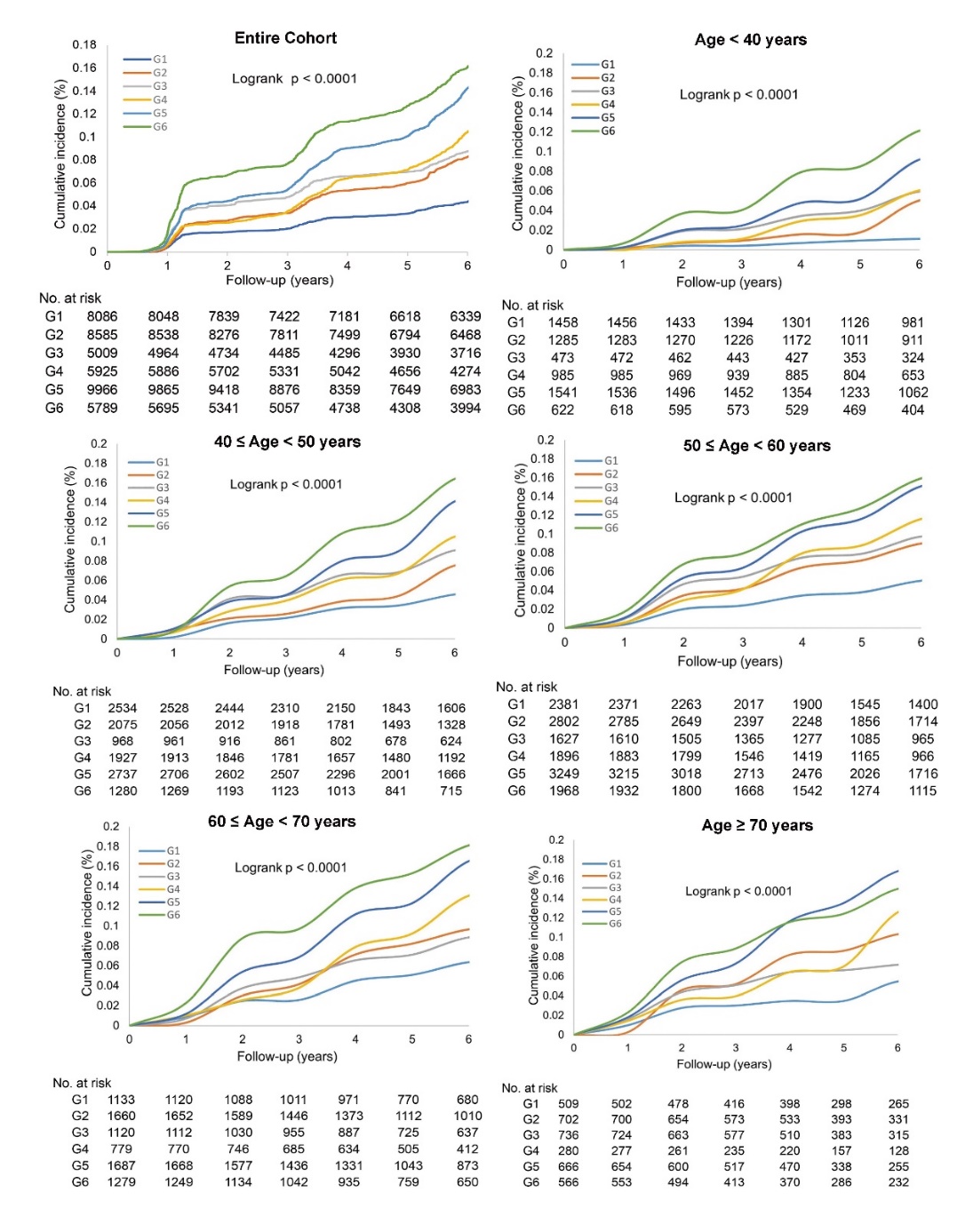
Fig. S3. Kaplan‒Meier curves of cumulative incidence of type 2 diabetes with the co-exposure subgroups in the entire cohort and across age subgroups

G1: CumAIP<-0.0699 & CumCRP<1 mg/L; G2: CumAIP<-0.0699 & 1 ≤CumCRP<3 mg/L; G3: CumAIP<-0.0699 & CumCRP≥3 mg/L; G4: CumAIP≥-0.0699 & CumCRP<1 mg/L; G5: CumAIP≥-0.0699 & 1 ≤CumCRP<3 mg/L; G6: CumAIP≥-0.0699 & CumCRP≥3 mg/L. G1 was used as the reference. Abbreviation: CumAIP: cumulative atherogenic index of plasma; CumCRP: cumulative high-sensitivity C-reactive protein.
